# Click Chemistry and Targeted Degradation: A Winning Combination for Medicinal Chemists?

**DOI:** 10.1002/cmdc.202300422

**Published:** 2023-09-14

**Authors:** Anna Pasieka, Eleonora Diamanti, Elisa Uliassi, Maria Laura Bolognesi

**Affiliations:** ^1^ Department of Pharmacy and Biotechnology Alma Mater Studiorum-University of Bologna Via Belmeloro 6 40126 Bologna Italy

**Keywords:** click chemistry, bioorthogonal chemistry, PROTACs, Targeted Protein Degradation, triazoles

## Abstract

Click chemistry is universally recognized as a powerful strategy for the fast and precise assembly of diverse building blocks. Targeted Protein Degradation (TPD) is a new therapeutic modality based on heterobifunctional small‐molecule degraders that provides new opportunities to medicinal chemists dealing with undruggable targets and incurable diseases. Here, we highlight how very recently the TPD field and that of click chemistry have merged, opening up the possibility for fine‐tuning the properties of a degrader, chemically assembled through a “click” synthesis. By reviewing concrete examples, we want to provide the reader with the insight that the application of click and bioorthogonal chemistry in the TDP field may be a winning combination.

## Graphical abstract

“Click chemistry” and targeted protein degradation – two flourishing trends in medicinal chemistry. May they be a winning combination? In this review, we provide the reader with selected examples offered by the combination of these two approaches trying to find a response to this question.

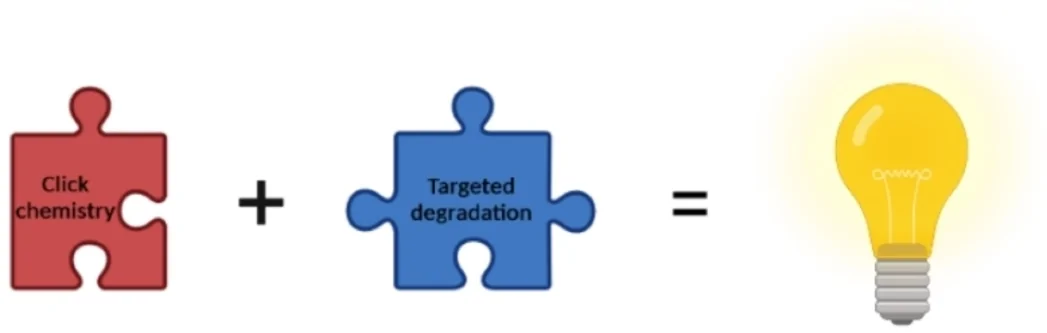

## 1 Introduction

Once a year the chemistry community stops research waiting for the announcement of its most prestigious award: the Nobel Prize. Last year, the Royal Swedish Academy of Sciences awarded The Nobel Prize in Chemistry 2022 to three great scientists: Carolyn R. Bertozzi, Morten Meldal and K. Barry Sharpless “for the development of click chemistry and bioorthogonal chemistry”. The choice was not surprising, when looking at recent publications’ trends in the field, where these two strategies are flourishing.

Prof. Sharpless and prof. Meldal independently reported the high‐yielded and regioselective copper‐catalyzed Huisgen cycloaddition reaction (Figure 1A), which now is recognized as the flagship of “click chemistry”.[^1^, ^2^] Few years later, the idea that similarly simple and fast reactions could be performed in living organisms^[3]^ was introduced by prof. Bertozzi, who performed the first “bioorthogonal reaction” in cells, i. e., the Staudinger ligation of azides with triarylphosphines.^[4]^ Over the years, these two methodologies, have completely changed the field of material science, but also chemical biology, medicinal chemistry, and even drug discovery.[^5^, ^6^, ^7^] In fact, these two new synthetic philosophies have become a perfect answer for the growing needs of pharma: in addition to the features mentioned above, click reactions satisfy the principles of green chemistry, such as prevention of hazardous waste and by‐products, minimized derivatization due to better selectivity and tolerance (insensitive to oxygen and water), atom economy, use of safer chemicals and solvents (environmentally benign), energy minimization (a lot of click reactions can occur with less or no heating conditions).

**Figure 1:**
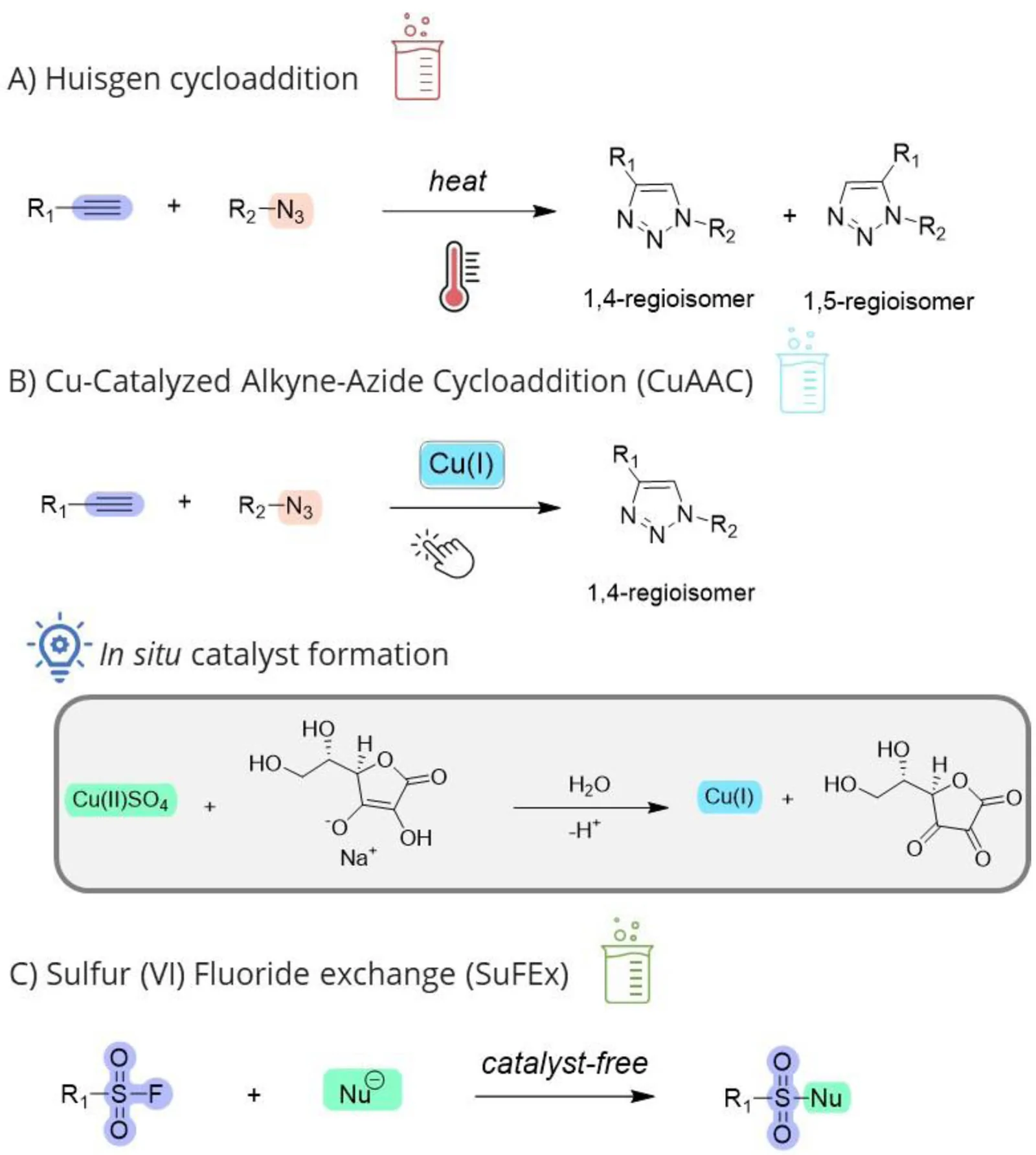
Click chemistry reactions: A) Huisgen cycloaddition reaction; B) Cu‐catalyzed azide‐alkyne cycloaddition (CuAAC); C) sulfur(VI) fluoride exchange (SuFEx).

The term “click chemistry” was first coined in 2001^[8]^ and referred to modular, wide in scope, stereospecific reactions in simple conditions giving a single product in very high yields after an easy isolation. The used reagents and solvents were requested to be commonly available, benign and easily removed (e. g., water).^[8]^ Some reactions were found to meet these requirements and included in the list: e. g., cycloadditions of unsaturated species (especially 1,3‐dipolar cycloaddition reactions), the Diels‐Alder family of transformations, nucleophilic substitution chemistry, and carbonyl chemistry of the “non‐aldol” type. However, the real breakthrough come from the discovery from both Sharpless and Meldal that copper(I) iodide substantially catalyzed the Huisgen 1,3‐dipolar cycloaddition reaction between azides and terminal alkynes forming 1,4‐regioisomers of 1,2,3‐triazoles.

Thanks to this discovery, the current gold standard of click chemistry is the Cu‐catalyzed azide‐alkyne cycloaddition (CuAAC; Figure 1B).^[9]^ In most applications, copper(II) sulfate is used as a catalyst together with a reducing agent – sodium ascorbate – to produce Cu(I) *in situ*. In contrast to the small diversity of catalysts, the reaction can be set up with a great variety of solvents, depending on the solubility of substrates. The most common choice is a mixture of solvents: water and miscible (sometimes just partially) organic solvents e. g., *tert*‐BuOH or different alcohols. Today, there are other popular examples of “click chemistry” reactions, e. g., thiol‐ene reaction, [4+1] cycloadditions between isonitriles (isocyanides) and tetrazines, which have been discussed in other recent reviews.[^10^, ^11^, ^12^]

The sulfur(VI) fluoride exchange (SuFEx) represents the most recent set of click chemistry transformations: a metal‐free click chemistry reaction, discovered by Sharpless in 2014.^[13]^ Specifically, SuFEx exploited the elevated reactivity of sulfur(VI) fluorides with appropriate nucleophiles, including amines (Figure 1C).[^13^, ^14^] Similar to CuAAC, SuFEx reactions have been reported to be highly efficient (high conversion), water friendly and easy to set up, while significantly differing from the previous one for harnessing metal catalyst‐free protocols. Moreover, the easy preparation and good properties of sulfonyl fluorides, such as the insensitivity to ambient oxygen and the hydrolytical stability, are other important advantages. Initially, sulfuryl fluoride (SO_2_F_2_) was used in form of gas, but, due to obvious drawbacks, over time it has been replaced with more stable, solid FSO_2_‐carrying reagents, such as fluorosulfuryl imidazolium salts.^[15]^ The most typical reactions revolve around the coupling of sulfonyl fluorides with silyl ethers or primary/secondary amines resulting in the formation of S−O bond or sulfonamides, respectively.[^16^, ^17^]

To note, the rapid formation of sulfonamides has particularly attracted the interest of medicinal chemists.^[18]^ Another intriguing aspect is that SuFEx technology can be exploited not only for creating connections, but also as for preparing reagents in their own right.[^19^, ^20^]

The concept of “bioorthogonal chemistry” is very similar to “click chemistry”, so that the two are often referred interchangeably. Yet, there is a crucial difference: for the first, the reagents are not toxic for living organisms and therefore, reactions can be performed *in vivo*.[^21^, ^22^] Three out of the most famous bioorthogonal transformations are (i) the Staudinger ligation[^4^, ^23^], (ii) the strain‐promoted azide‐alkyne cycloaddition (SPAAC), also called as “Cu‐free click chemistry”[^21^, ^24^] and (iii) inverse electron demand Diels‐Alder (IEDDA) cycloaddition (Figure 2).[^25^, ^26^, ^27^] The Staudinger reaction is the first reported bioorthogonal reaction between an azide and a phosphine, resulting in an amide.[^4^, ^28^] In turn, SPAAC reaction was developed by Bertozzi to eliminate the cytotoxic effect of copper *in vivo*. Instead of using the catalyst, the alkyne moiety is introduced in a strained difluorooctyne (DIFO), dibenzocyclooctyne (DBCO) or biarylazacyclooctynone (BARAC). These scaffolds allowed for the activation of the alkyne fragment and thus, the formation of the triazole ring.[^22^, ^24^, ^29^] IEDDA is believed as the fastest bioorthogonal reaction (Figure 2). It is performed between tetrazine and trans‐cyclooctene (TCO) derivatives and, since its description by Blackman, is successfully used for many biomedical applications.[^25^, ^26^, ^27^, ^30^]

**Figure 2:**
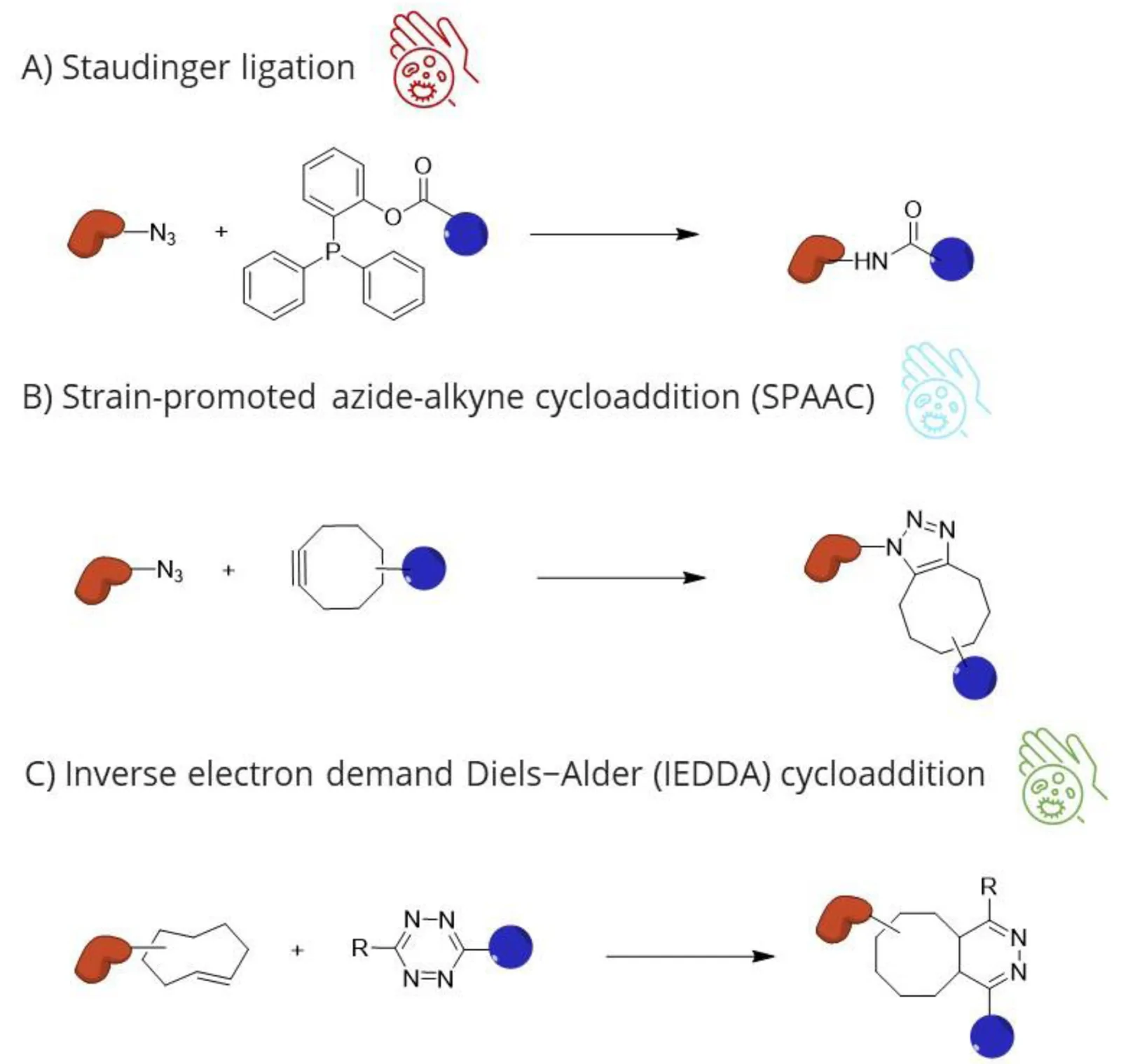
Bioorthogonal chemistry transformations: A) Staudinger ligation; B) strain‐promoted azide‐alkyne cycloaddition (SPAAC); C) inverse electron demand Diels‐Alder (IEDDA) cycloaddition.

The simplicity, speed, ease, and wide scope of click/ bioorthogonal chemistry have been so much appreciated that both strategies are widely used in the search of many drugs, e. g., anticancers, antivirals, peptidomimetics, antiparasitics or anti‐inflammatories.[^31^, ^32^]

As highlighted by the Nobel Committee,^[33]^ thanks to its modular nature and robustness, the principle may be equated to an “*IKEA* ‘flatpack’ approach, in which all necessary components, the ‘building blocks’, along with a set of easy‐to‐use hardware, the ‘reactions’, were provided together with a reliable assembly instruction for almost anybody to follow”. On this basis, it is intuitive that click chemistry is particularly powerful in the synthesis of heterobifunctional molecules, i. e., molecules consisting of two protein‐binding moieties joined *via* a linker of appropriate length.^[34]^ This particularly applies to degraders, an innovative class of heterobifunctional agents that have been recently brought to the forefront in medicinal chemistry.[^35^, ^36^, ^37^]

There are different groups of degraders: PROTACs – proteolysis targeting chimeras, LYTACs ‐ lysosome targeting chimeras,^[38]^ AUTACs – autophagy targeting chimeras,[^39^, ^40^] hydrophobic tagging (HyT) and RIBOTACs.[^41^, ^42^, ^43^, ^44^, ^45^, ^46^] They all consist of two *‘building blocks’*: a ligand binding the target of interest and a moiety recruiting the target responsible of inducing degradation, connected *via* a suitable linker (Figure 3).

**Figure 3:**
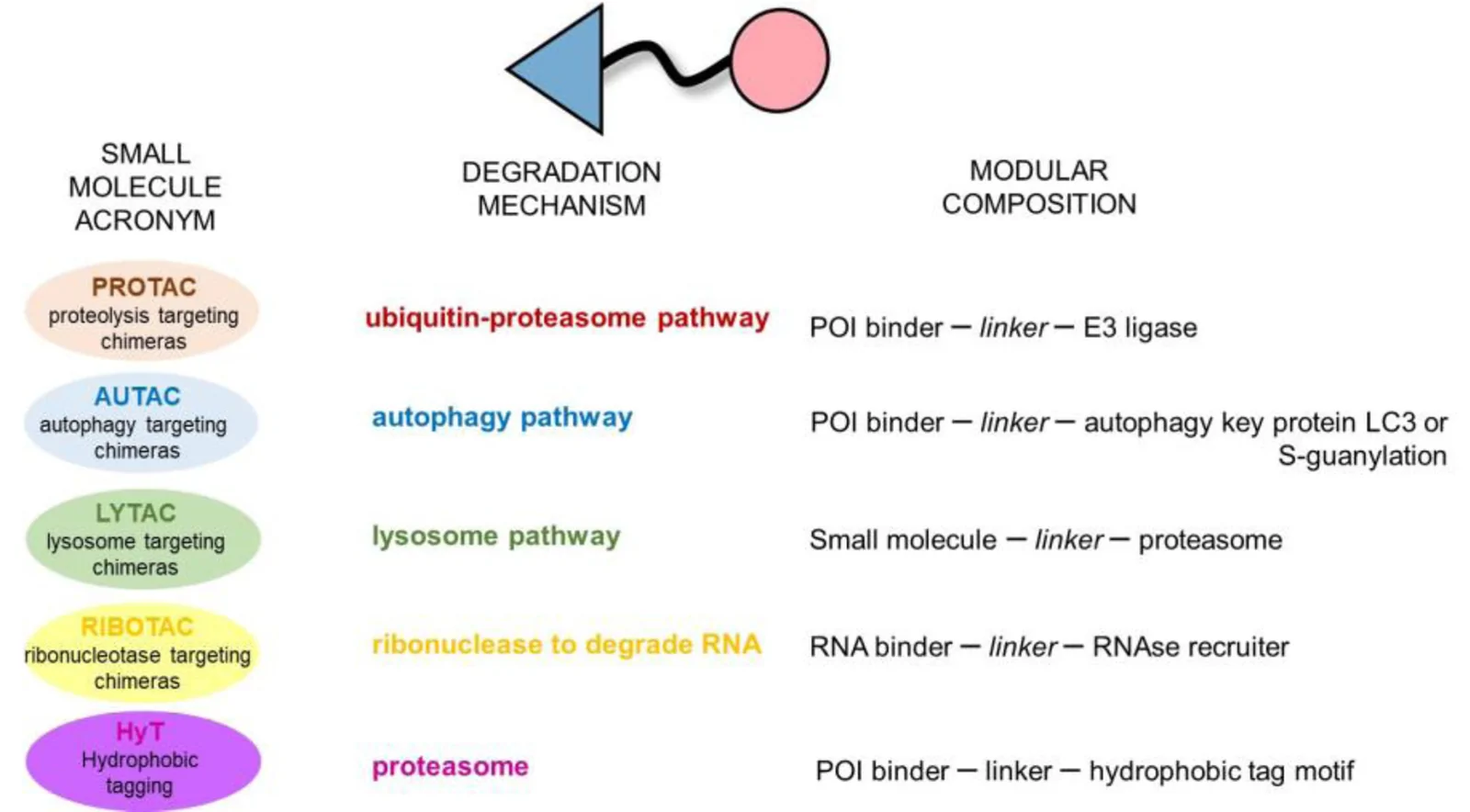
Groups of heterobifunctional degraders, together with their mechanism of degradation and modular composition.

Specifically, PROTACs, featuring a ligand for the protein of interest (POI) joined with a spacer to an E3 ubiquitin ligase binder, promote POI ubiquitination through the formation of the ternary POI‐PROTAC‐E3 ligase complex, and the consequent selective degradation by the proteasome. It is clear that linker length, composition and rigidity are all crucial for creating a productive ternary complex and, consequently, for inducing the degradation process.[^47^, ^48^, ^49^] As a result, the selectivity, ease, rapidity, and modularity of click ligations make them nearly ideally suited for the construction of degraders, a process that otherwise involves multi‐step protocols and difficult purifications. These are very tedious tasks and performing successful linker chemistry, i. e., allowing the combination of the different recruiting elements in a biologically benign, fast, and selective fashion, remains a complex endeavor. Conversely, click chemistry may enable rapid structure‐activity relationship (SAR) profiling, ensuring a fast exploration of the chemical space in terms of ligand assembly and probing different attachment points, as well as linker length.

However, it was not only the simplicity and speed of click and bioorthogonal chemistry that have attracted great interest for the development of new degraders. The introduction of the 1,2,3‐triazole ring into a linker could change the physicochemical properties of the molecule in terms of topological polar surface area (TPSA) and lipophilicity (distribution coefficient, logD and *c*LogP) and hence, improve cell permeability, solubility or stability.[^50^, ^51^] Moreover, a triazole ring increases the rigidity of the linker, potentially allowing the proper proximity of POI and E3 ligase to form an effective ternary POI‐PROTAC‐E3 ligase complex.[^52^, ^53^] In turn, harnessing bioorthogonal chemistry may overcome problems related to PROTAC delivery into the cells (mainly due to their high molecular weight). This is achieved through the delivery of smaller POI‐ and E3 ligase‐ligand precursors, which can react directly in the cells providing the final degraders, termed CLIPTACs (i. e., in‐cell click‐formed proteolysis‐targeting chimeras).[^50^, ^54^]

Herein, it is our goal to highlight the most interesting, exciting, and useful points of intersection between click chemistry and degrader discovery, with a particular emphasis on PROTACs. Given the boom of this topic, the current review will focus on selected examples that aim to highlight the growing impact and potentialities offered by the combination of these two approaches.

## 2 Cu‐Catalyzed Azide‐Alkyne Cycloaddition (CuAAC)

### 2.1 “Click chemistry” and targeted degradation: the debut

In 2017, two back‐to‐back articles were published in a *J. Med Chem* Special issue dedicated to “Inducing Protein Degradation as a Therapeutic Strategy”, which marked the debut of CuAAC in PROTAC synthesis.^[55]^ Jung's group synthetized triazole‐based PROTACs targeting sirtuins (Sirt)^[56]^ – a family of histone deacetylases, involved in the pathogenesis of many diseases.^[57]^ The starting point was the Sirt2‐selective and potent SirReal, selected as the POI ligand,^[58]^ and the well‐known cereblon (CRBN) ligand thalidomide – chosen as E3 ligase binder. The final coupling of the alkynylated derivative of the POI ligand with a thalidomide‐derived azide was performed in water: *tert*‐BuOH (1 : 1) mixture by addition of copper sulphate and sodium ascorbate. The reaction was carried out at 60 °C for the first hour, and then at room temperature for 16 hours. The first‐in‐class triazole‐based PROTAC (**1**) (Figure 4) was obtained in 33 % yield. For the first time, **1‐** induced Sirt degradation demonstrated that the exploitation of a triazole linkage had the potential of accelerating the synthesis of novel degraders and inducing the degradation by creating the ternary complex.^[56]^

**Figure 4:**
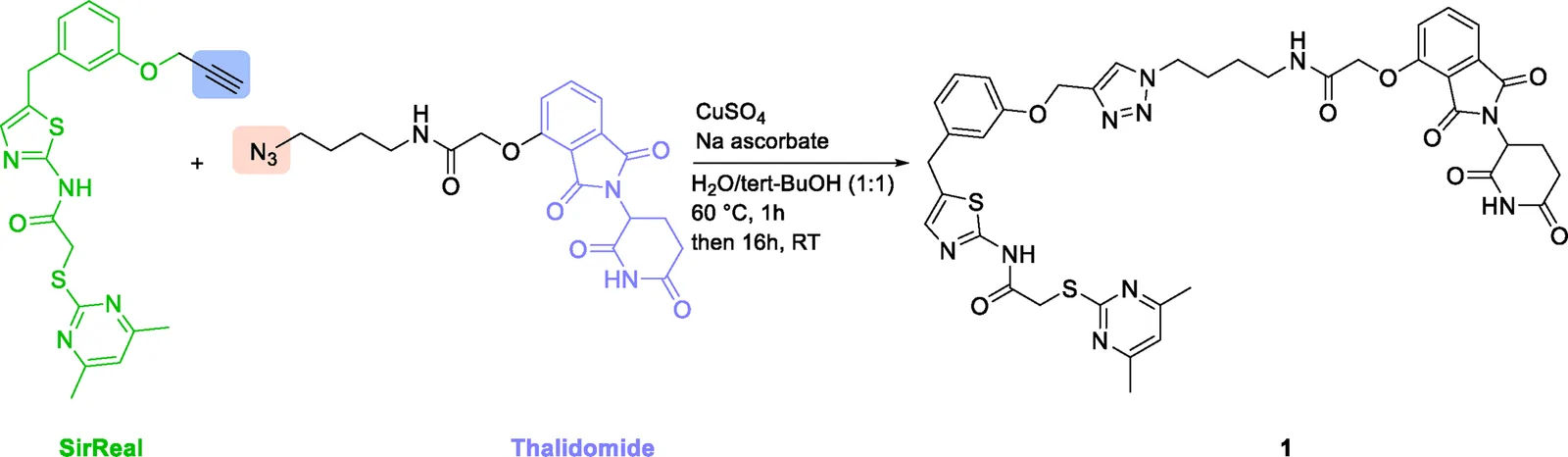
Synthesis of first‐in‐class triazole‐based PROTAC (**1**) *via* CuAAC.

Intriguingly, in the same special issue Wurz *et al*. proposed the first “click chemistry platform” to obtain rapidly and efficiently novel PROTACs by CuAAC.^[59]^ To validate this breakthrough idea, the authors focused on PROTACs directed to bromodomain BRD4. As the POI ligand, the well‐known JQ‐1 moiety binding to BRD4^[60]^ was converted from a *tert*‐butyl ester to an azido derivative by reaction with 2‐azidoethanamine. As E3 ligase binders, VHL and CRBN ligands functionalized with various PEG linkers terminating with alkyne functionalities were prepared. The typical CuAAC reaction conditions (CuSO_4_, sodium ascorbate, 0.1 M in THF/water, room temperature, 3 hours) were used to obtain ten final compounds (**2**–**11**) in optimal yields (55–90 %) (Figure 5). Proximity and protein degradation assays proved the ability of the triazole‐based PROTACs **2**–**11** to create the ternary complex and thus, to induce the proteasome mediated degradation.^[59]^

**Figure 5:**
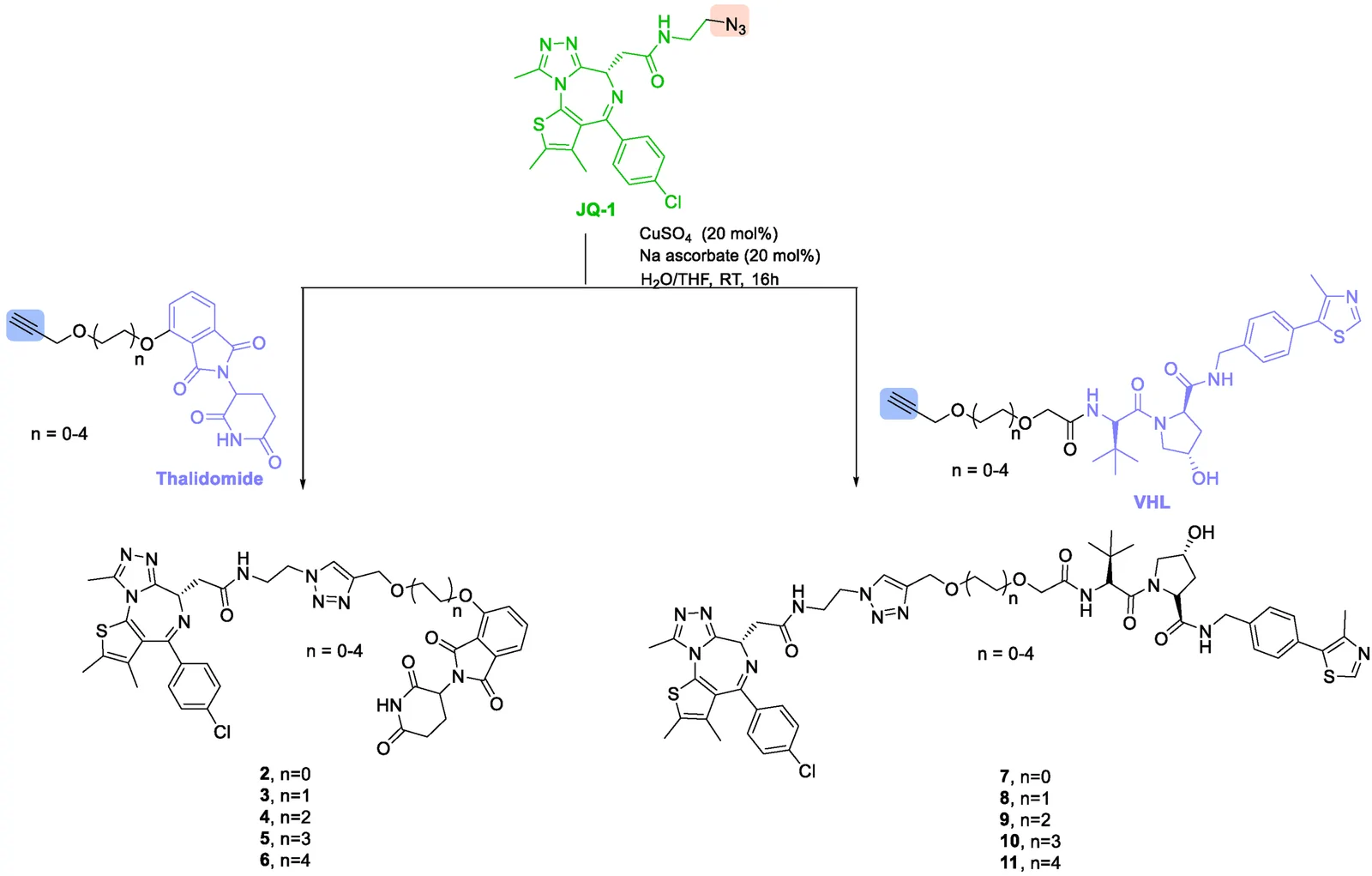
Click chemistry platform to obtain a library of novel PROTACs (**2**‐**11**).

### 2.2 CuAAC reaction in the synthesis of new PROTACs

A similar synthetic procedure was used in the optimization of first‐in‐class cyclin‐dependent kinase 2 (CDK2) selective degraders.^[61]^ As the POI ligand, CDK2 inhibitor J2 was selected (Figure 6). In turn, thalidomide was used as the E3 ligase binder. First, CDK2 binders with terminal alkyne group were prepared. Then, to optimize the linker length, various azide‐terminated PEG‐thalidomide were synthetized. Eventually, using CuAAC reaction (CuSO_4_, sodium ascorbate, DMF/water, 70 °C, 4 hours) both fragments were rapidly combined to provide four final compounds with yields ranging from 30 to 60 %. The most promising CDK2 degrader (**12**) showed not only the selective degradation of CDK2 protein but was also effective in acute myeloid leukemia (AML) primary cells. Therefore, triazole‐based PROTAC **12** demonstrated a potential application in AML.[^61^, ^62^]

**Figure 6:**
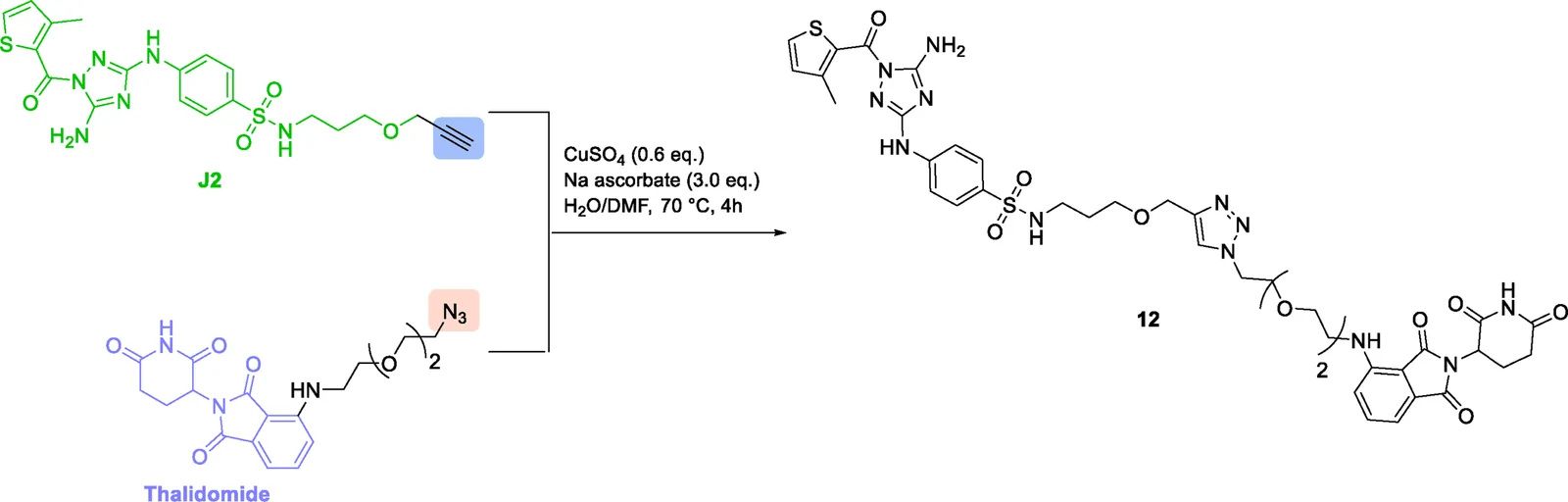
Synthesis of CDK2 selective PROTAC **(12)** *via* CuAAC.

Fuchs and coworkers decided to examine the impact of triazole position within various linker on physicochemical and biological properties of novel CDK9 degraders.^[63]^ Pan‐CDK9 inhibitor AT7519 was selected as the POI ligand^[64]^ and thalidomide as the E3 ligase binder. The series of ten PROTACs containing triazole‐alkyl linkers of different length (**13**–**22**, Figure 7) were synthetized using CuAAC reaction (CuSO_4_, sodium ascorbate, THF/water, RT, above 20 h) with yields ranging from 18 to 100 %. Then, their degradation activity, kinetic solubility and lipophilicity were evaluated. The data confirmed that compounds with greater cell permeability have higher *in vitro* biological activity. Of note, while keeping constant the linker length between the POI and E3 ligase ligands, the proximity of the triazole ring to the piperidine or benzene ring significantly influenced the lipophilicity and aqueous solubility. It is important to highlight that the relationship between degradation activity and physicochemical properties is not always fully clear. PROTAC permeability seems to be an important factor, but not crucial to create a ternary complex and induce POI degradation. Nevertheless, this research has showed that slight differences in the structure and the use of click chemistry may help in improving drug‐likeness of novel degraders, already at the early stage.^[63]^

**Figure 7:**
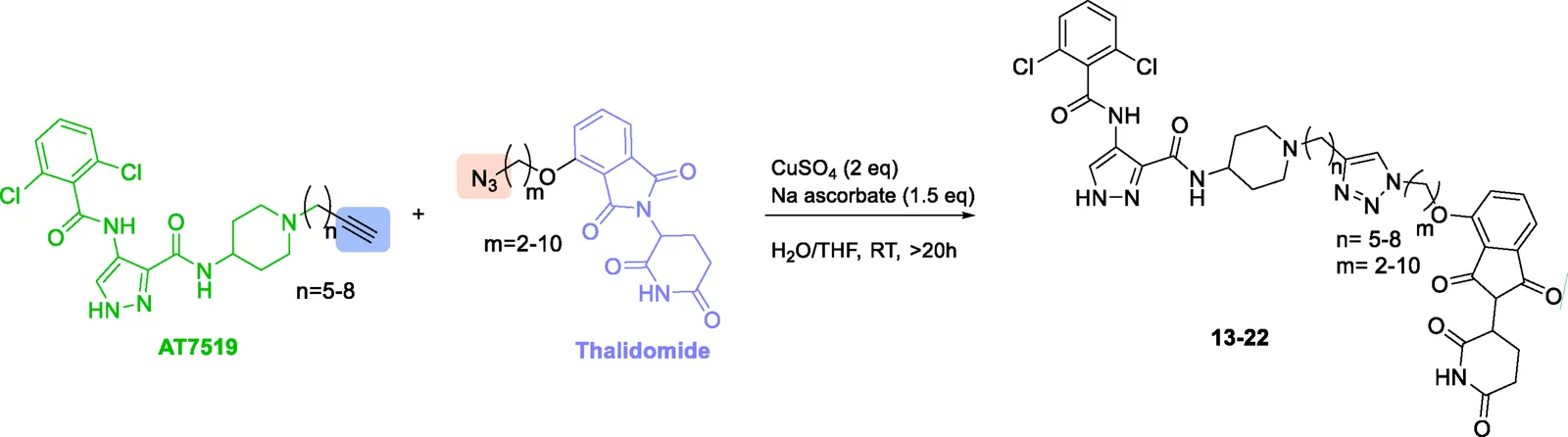
Synthesis of CDK9‐directed PROTACs (**13**‐**22**) *via* CuAAC.

Thanks to its effectiveness, the Wurz's strategy^[59]^ was applied in the synthesis of PROTACs targeting glycogen synthase kinase 3 (GSK‐3β) for the treatment of Alzheimer's disease (AD). To explore the potential degraders, the well‐known GSK‐3β inhibitor AZD2858 was used. In turn, as E3 ligase binders, a library of different CRBN (pomalidomide, lenalidomide) and von Hippel‐Lindau (VHL) ligands were used. More than thirty triazole‐based PROTACs were obtained in a click reaction (CuSO_4_, sodium ascorbate, a mixture of water/*tert*‐BuOH/DCM/DMF, RT) with yields ranging from 20 to 80 %. Among them, PT‐65 (**23**) (Figure 8) was selected as the most promising agent with degradation effect in the nanomolar range and a positive outcome in an *in vivo* AD model.^[65]^ The SAR study showed that the linker featuring PEG and triazole was the optimal for the biological activity. Moreover, with the increasing length of the linker, the activity was reduced. It was observed that the physicochemical properties of PROTACs played a key role in achieving the desired effect – three of the most active molecules showed similar logP, bioavailability and membrane permeability.^[65]^ This was not the only attempt to the search for new degraders in neurodegenerative diseases. Other series of GSK‐3β PROTACs based on the potent pyridinethiazole inhibitor was reported.[^66^, ^67^] Among these compounds, the triazole linker turned out to be a key spacer to form a productive ternary complex and thus, to induce the GSK‐3β degradation.^[66]^

**Figure 8:**
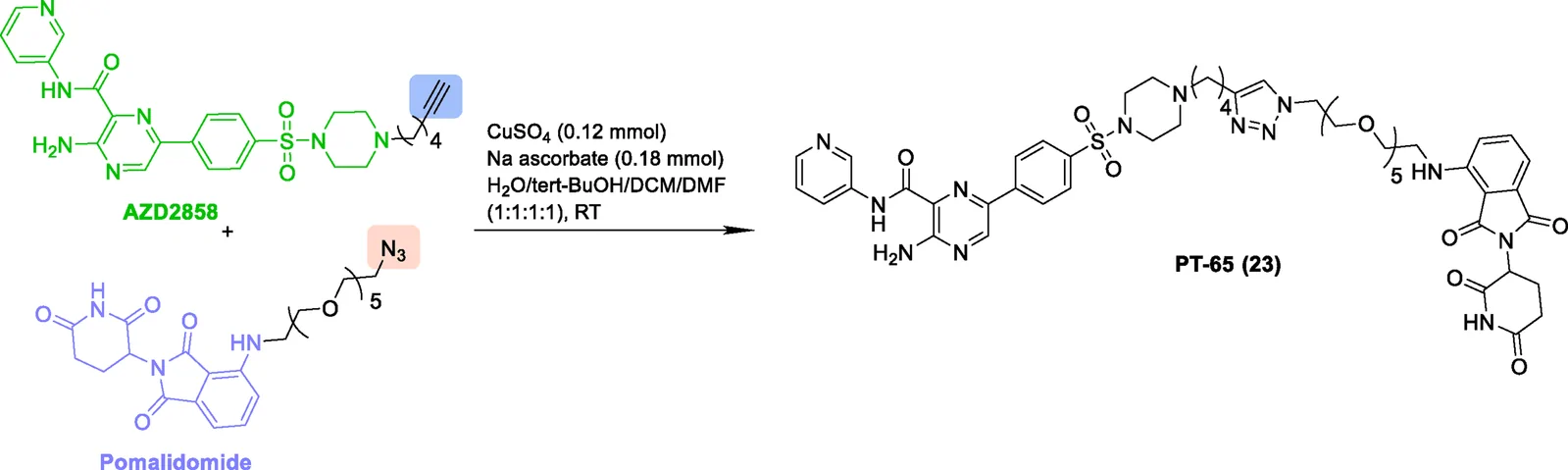
Synthesis of GSK‐3β ‐directed PROTAC **23** *via* CuAAC.

A slightly different condition of CuAAC reaction was used in the preparation of histone deacetylase 6 (HDAC6) degraders.^[68]^ HDAC6 is a protein responsible for deacetylation of some cytoplasmic proteins and its dysregulation is involved in cancer development.^[69]^ Yang and colleagues designed and synthetized four PROTACs consisting of the crebinostat structure (POI ligand)^[70]^ and thalidomide moiety connected *via* different triazole‐PEG linkers. The click reactions were performed in a mixture of water/*tert*‐BuOH with the addition of a complexing ligand ‐ tris[(1‐benzyl‐1*H*‐1,2,3‐triazol‐4‐yl)methyl]amine (TBTA) in good yields (60‐70 %). The use of TBTA accelerates the CuAAC reaction by maintaining the Cu(I) oxidation state and avoiding the oxidation of catalytic Cu(I) by dissolved oxygen.^[71]^

The aldehyde handle on the linker was then exploited for the condensation with the “HDAC biasing” acyl hydrazide reagent (Figure 9A). Compound **24** **a** with a 3‐PEG linker displayed the highest degradation effect (Figure 9B).^[68]^ Further research resulted in the development of a second‐generation HDAC6 degraders with improved potency and selectivity. The most potent compound (**24** **b**) (Figure 9B) degraded HDAC6 at a nanomolar concentration and showed antiproliferative effect in multiple myeloma cells.^[72]^ Munoz *et al*. synthetized just one niclosamide based anti‐cancer PROTAC *via* a CuAAC protocol. The PROTAC consists of a POI ligand, anthelmintic drug niclosamide, connected by a linker to the E3 ligase binder VH‐032. Compared to the previously described conditions, the authors used different combination of reagents and solvents: copper iodide, hexamethylphosphoramide (HMPA) and *N*,*N*‐diisopropylethylamine (DIPEA), resulting in 38 % yield. The addition of DIPEA accelerated copper(I)‐acetylide formation, and thus, increased reaction rates.^[71]^ Then, an amide coupling with a VHL ligand was performed, leading to the final compound **25** (Figure 10). Despite the positive *in vitro* antiproliferative results, for **25** the degradation activity was not confirmed.^[73]^ This example, clearly demonstrates that the introduction of a triazole ring *via* click chemistry cannot be a one‐size‐fits‐all‐solution. On the other hand, CuAAC were profitably applied in the development of PROTACs targeting, among others, estrogen receptor alpha (ERα),^[74]^ Fms‐like tyrosine receptor kinase 3 (FLT3),^[75]^ or cyclin‐dependent kinase 6 (CDK6).^[76]^

**Figure 9:**
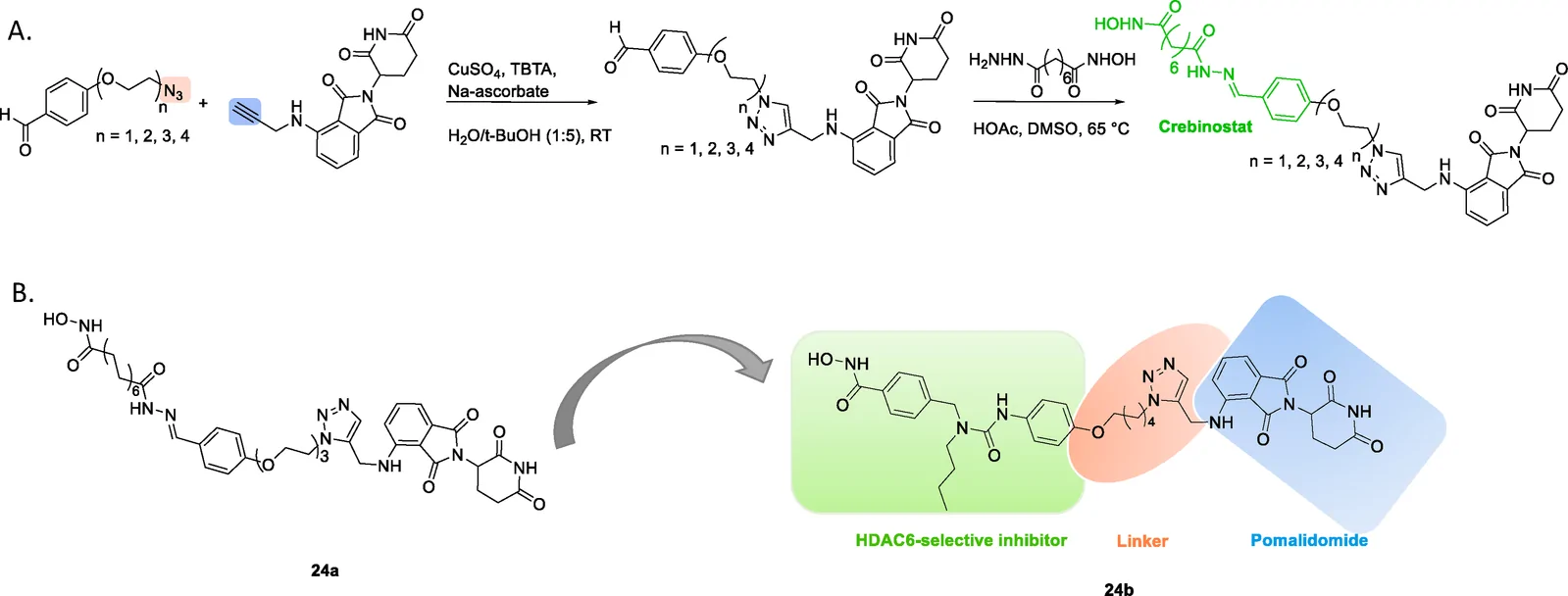
A) Synthesis of HDAC6‐directed PROTACs *via* CuAAC; **B)** Design of second‐generation HDAC6‐directed PROTAC **24** **b** from **24** **a**.

**Figure 10:**
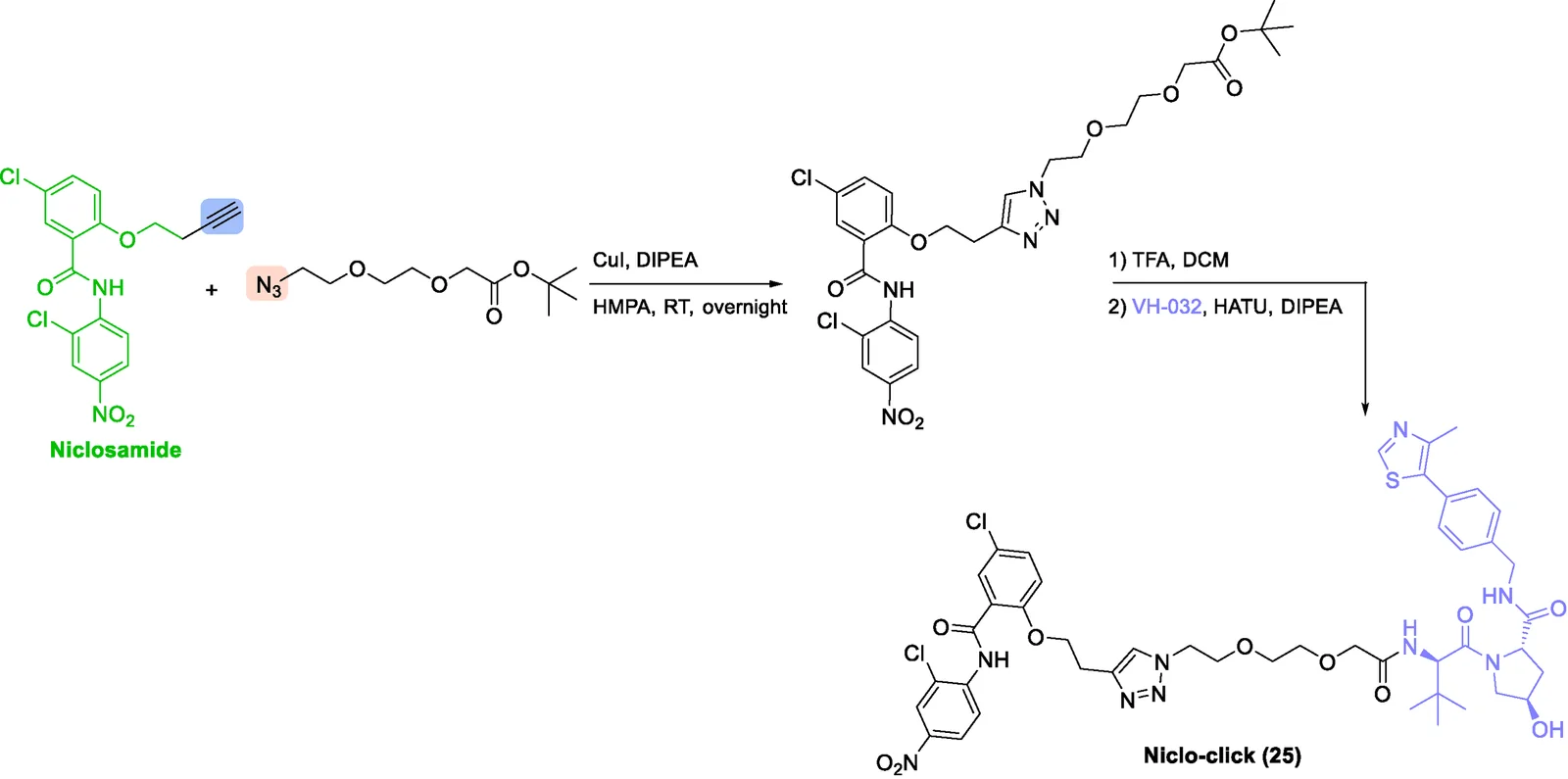
Synthesis of Niclo‐click PROTAC **25** *via* CuAAC.

### 2.3 Dual PROTACs

The implementation of “click chemistry” has contributed to expand the research scope of PROTACs to new modalities, leading to the development of so‐called *multitarget* PROTACs, i. e., single molecules able to recruit and degrade multiple target proteins.^[43]^ Based on this, dual triazole‐based PROTACs targeting simultaneously the epidermal growth factor receptor (EGFR) and poly (ADP‐ribose) polymerase (PARP), have been developed.^[77]^ The two POI warheads, namely gefitinib and olaparib, were combined *via* trifunctional natural amino acids (as star‐type core linkers) with the respective E3 ligand, *via* a CuAAC protocol (Figure 11). This convergent synthetic strategy (CuSO_4_, sodium ascorbate, water/THF, RT, maximum 3 h) resulted in the efficient development of eight dual PROTACs, including four thalidomide‐based and four VHL ligand‐based PROTACs, with yields ranging from 20 to 80 %. PROTAC **26** turned out to be the most active of the series, allowing the simultaneous degradation of both EGFR and PARP targets in cancer cells.^[77]^

**Figure 11:**
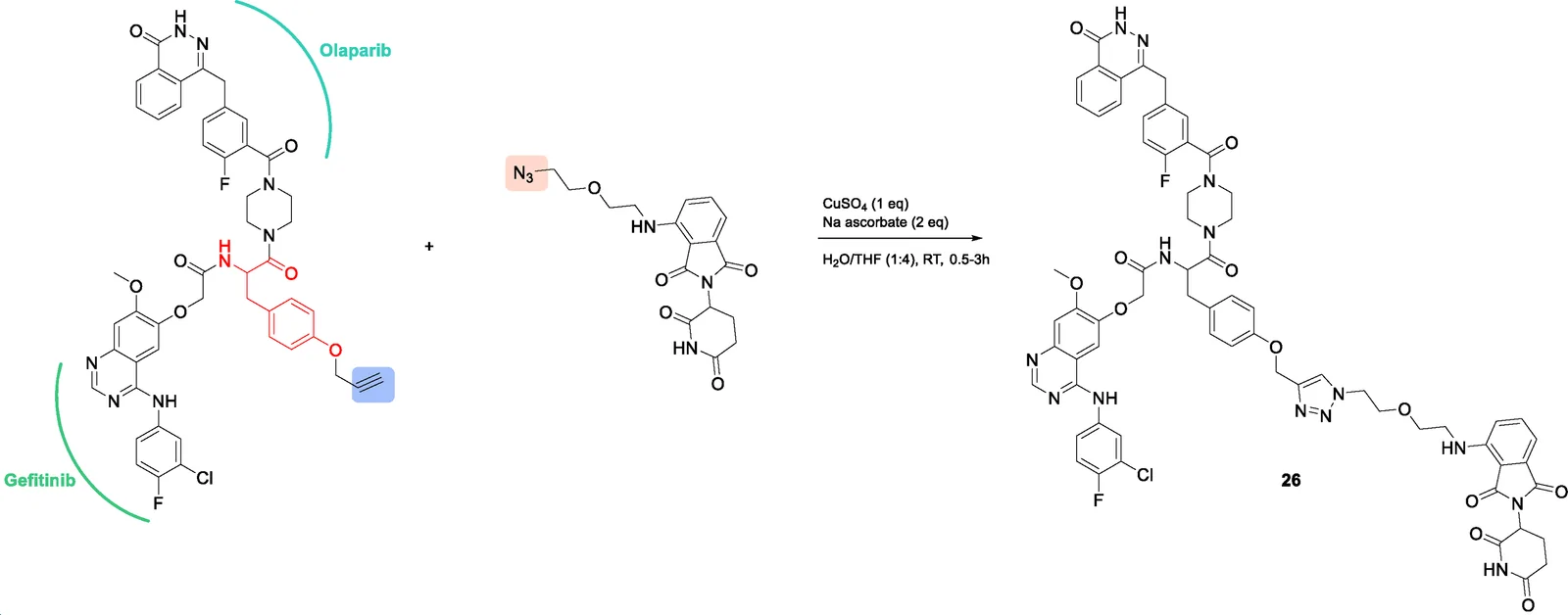
Synthesis of dual EGFR/PARP PROTAC **26** via CuAAC. The star‐type core linker is highlighted in red.

### 2.4 Hydrophobic tagging (HyT) via click chemistry

The CuAAC was also successfully exploited for the development of hydrophobic tag‐based degraders. The hydrophobic tagging (HyT) builds on the idea that the introduction of fragments mimicking a partially denatured protein folding state into a targeted degrader, can induce the chaperone‐mediated degradation by the proteasome.^[78]^ According to this strategy, Xie *et al*. proposed two series of selective androgen receptor degraders (SARDs) bearing a triazole‐based linker.^[79]^ As the POI ligand, the AR agonist RU59063 was chosen,^[80]^ linked to an adamantyl moiety as a well‐known hydrophobic tag (HyT39).^[81]^ These two fragments were connected *via* PEG, alkyl or aromatic linkers of different lengths using CuAAC reaction (CuSO_4_, sodium ascorbate, water/THF, RT, 0–2 h). Twenty‐nine final compounds were obtained, with yields ranging from 46 to 86 %. The most active compound **27** (Figure 12) displayed high AR binding affinity, good inhibitory activity against LNCaP prostate cancer cell line, and promising AR degradation effect.^[79]^ The positive degradation effect showed that triazole‐based linkers can be used for the synthesis of different type of degraders.

**Figure 12:**
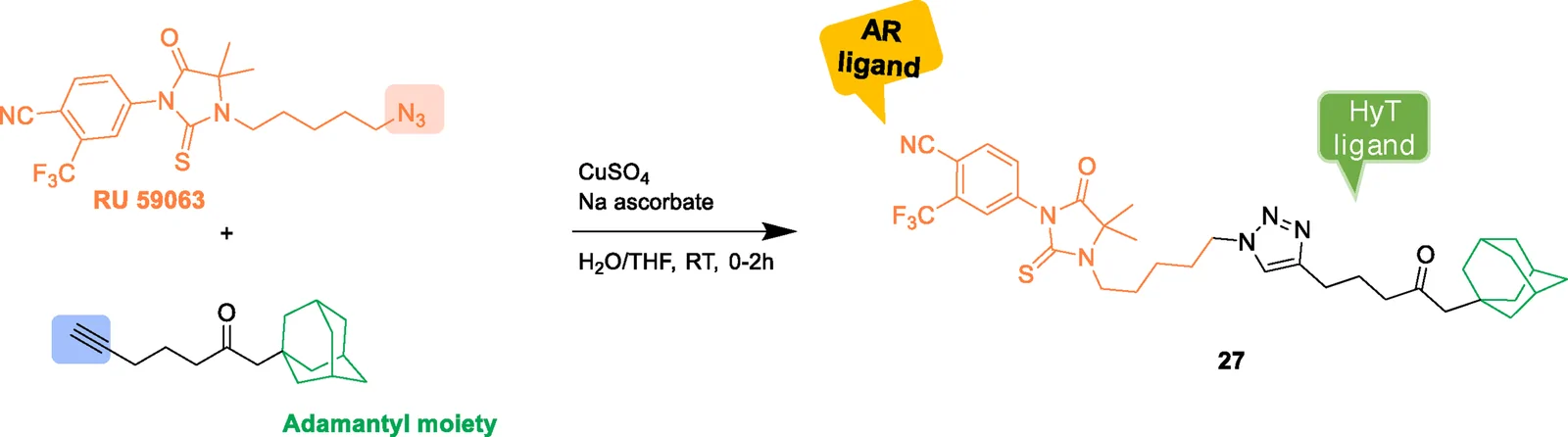
Synthesis of selective androgen receptor hydrophobic tag‐based degrader **27** *via* CuAAC.

### 2.5 Oligonucleotide‐type click chemistry

Noteworthy, click chemistry is not only used in the synthesis of PROTACs featuring small molecules as POI ligands. It has also been successfully applied to assemble oligonucleotide, further confirming its versatility and wide scope. Naganuma *et al*. reported a series of oligonucleotide‐type ERα degraders,^[82]^ synthesized *via* a CuAAC protocol. A decoy oligonucleotide able to bind to ERα receptor was first selected as POI ligand. Then, the alkyne‐terminated decoy moiety (**ER(dec)**, Figure 13) was connected *via* triazole linkers with different azide‐terminated E3 ligase ligands: LCL161 – inhibitor of apoptosis protein ligand (IAP) (**LCL‐PEG3‐N3**), VH032 – ligand of von Hippel Lindau protein, and pomalidomide – CRBN ligand.^[83]^ The click reactions were performed in a mixture of common solvents (DMSO, methanol, water) with a catalyst in form of copper(I) salt (iodocopper(I) triethyl phosphite, CuI•P(OEt)_3_) and triethylamine (TEA) addition at 40 °C. Implementing directly a copper(I) salts excludes the necessity of the use of reducing agents, but the copper(I) needs to be stabilized by bulky organic substituents, like triethyl phosphite.^[71]^ The addition of TEA increased the reaction rate.^[71]^ The most promising molecule in terms of highest ERα degradation effect was **LCL‐ER(dec)** (**28**, Figure 13) consisting of the LCL161 moiety, a PEG3 linker and the ERα‐binding decoy ER(dec).^[82]^

**Figure 13:**
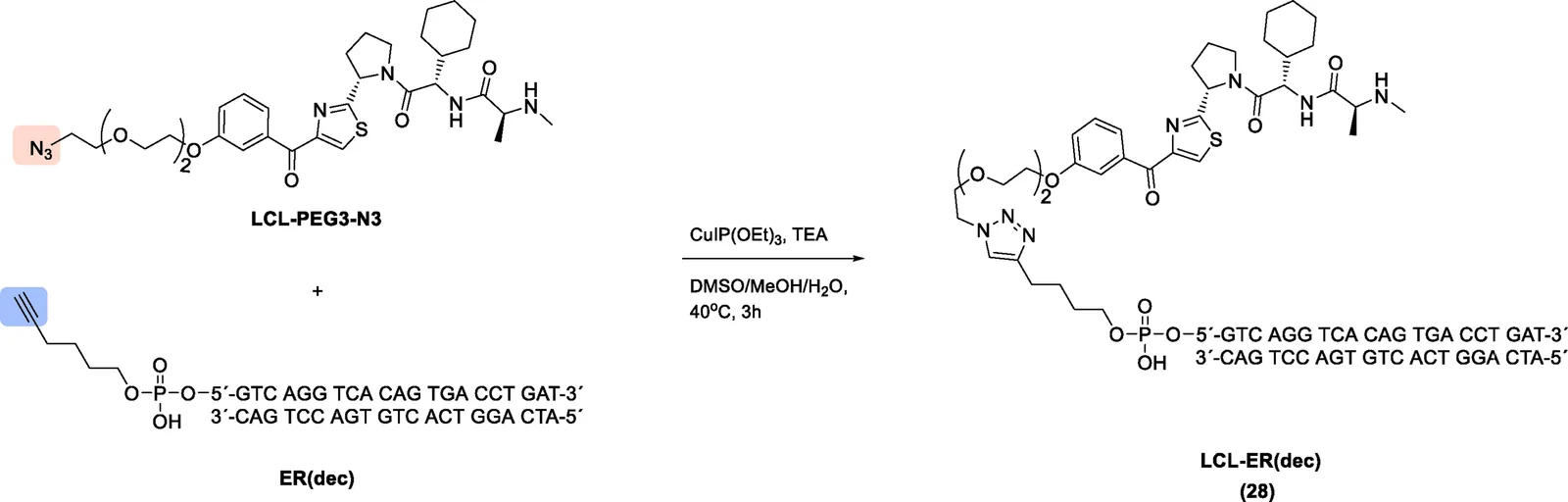
Synthesis of LCL‐ER(dec) (**28**) via CuAAC.

Similarly, another class of ERα degraders,^[84]^ featuring the specific estrogen response element (ERE) directed to the DNA‐binding motif of ERα was developed.^[85]^ The reaction between ERE and VHL‐based E3 ligase was performed by CuAAC reaction and the obtained ERE‐PROTAC displayed a satisfactory degradation effect in breast cancer cells.^[84]^ These two examples highlighted the versatility of click chemistry, expanding its use to the development of non‐small molecule‐based degraders.

### 2.6 DNA‐encoded library screening

The use of click chemistry in the TPD field has been successfully employed in combination with emerging drug discovery technologies. Disch *et al*. proposed its use in the search of new bispecific ERα degraders,^[86]^ starting from DNA‐encoded chemical library (DECL) screening against ERα.^[87]^ DECL is a technology that allows to synthetically generate millions of compounds in a rapid way and, to screen them against the selected biological target. In fact, the synthetized compounds are connected to DNA fragments which work like “bar codes”, allowing a fast hit identification.^[87]^ In the PROTAC field, this is particularly important, not only for the rapid identification of novel POI binders, but also because it allows to know the linker attachment point a priori, given the connected DNA fragment. Among the DECL, two promising molecules were selected as hits, leading to the identification of the ERα ligand, which was transformed into the corresponding azide derivative. Click chemistry synthesis between azide‐derivatized ERα ligand and an alkyne‐terminated E3 binder panel rapidly generated ERα degraders.^[86]^ The synthetic strategy involved the use of CuSO_4_, sodium ascorbate, THPTA, water/DMSO, RT. To note, THPTA ‐ tris((1‐hydroxy‐propyl‐1H‐1,2,3‐triazol‐4‐yl)methyl)amine, is a ligand exploited in CuAAC, which, similar to the previously mentioned TBTA, accelerates the cycloaddition by maintaining the Cu(I) oxidation state. The advantage of employing THPTA compared to TBTA is its water solubility.^[71]^ Thanks to the combination of DECL technology and click chemistry, Erα‐targeted PROTAC **29**, with beneficial *in vivo* effects, was discovered (Figure 14).^[86]^ In addition, this work established that DECL information can be directly leveraged to efficiently produce PROTACs starting from an azide derivative of the POI ligand obtained by replacing the DNA tag, and then cross‐reacted with a series of E3 binder alkynes, utilizing click chemistry.

**Figure 14:**
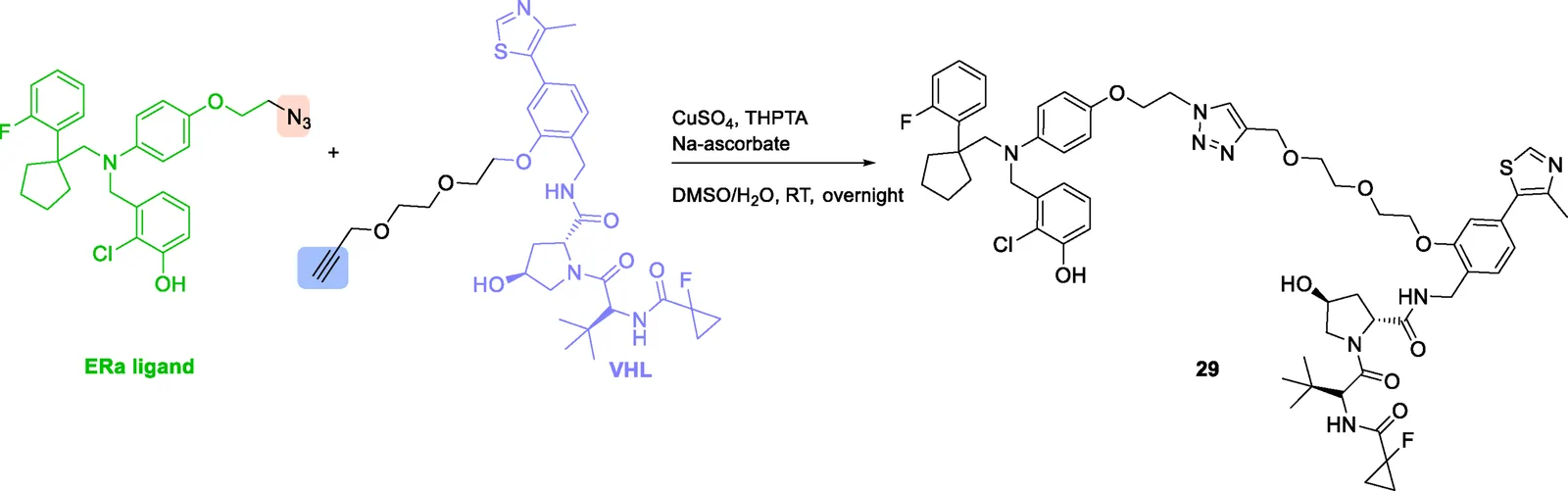
Synthesis of bispecific ERα degrader **29** *via* CuAAC.

### 2.7 Solid‐phase organic synthesis in combination with click chemistry

Another interesting idea proposed by Xu *et al*. is the combination of solid‐phase organic synthesis (SPOS)^[88]^ with click chemistry for the development of new PROTACs.^[89]^ Because of a difficult purification due to the highly polar character of the thalidomide scaffold, SPOS was proposed as a suitable solution.^[90]^ To perform CuAAC reaction, a supported azide reagent was prepared by linking an azide‐terminated pomalidomide to the resin *via* an amine bond. In the next step, it was reacted with an alkyne‐terminated POI ligand (TFC‐007) under standard reaction conditions (CuI, DIPEA, DMF, RT), providing, after acidic resin cleavage, the triazole‐based PROTAC **30** with 99 % purity, but very poor yield (1 %) (Figure 15). PROTAC **30** was found to have the highest degradation activity against hematopoietic prostaglandin D synthase (H‐PGDS) with respect to the corresponding amide‐ and urea‐based PROTACs. Although further optimization is required, once more, click chemistry combined with SPOS might facilitate the rapid development of PROTACs directed to different POIs.^[89]^

**Figure 15:**
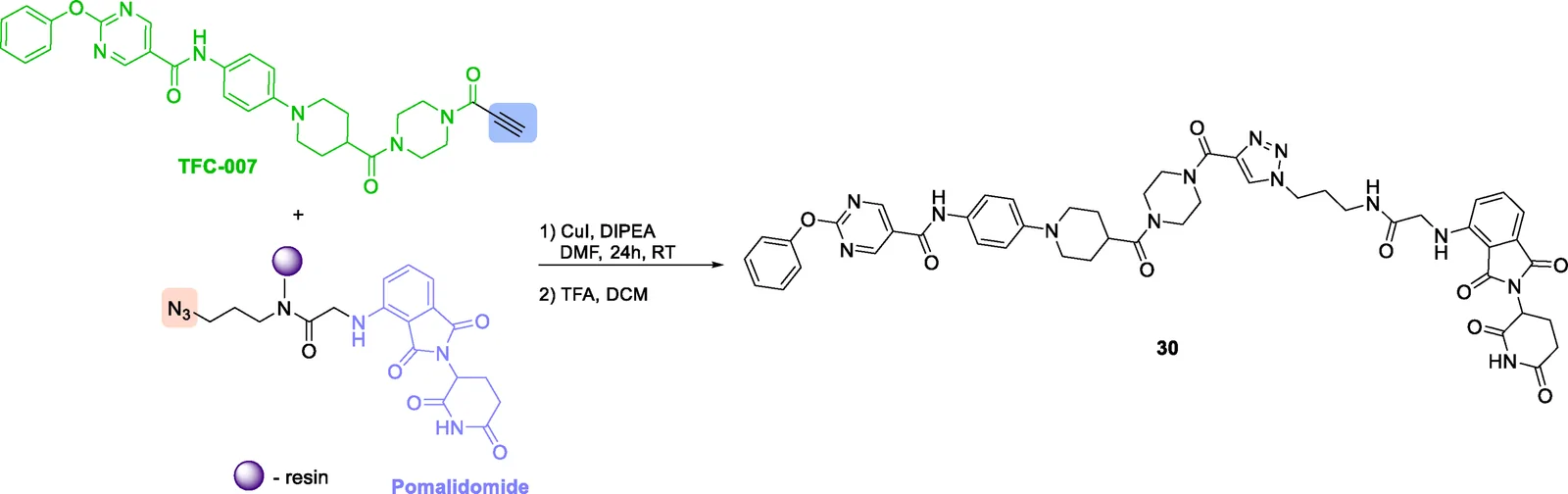
The synthesis of triazole‐based PROTAC (**30**) *via* a solid‐phase CuAAC

## 3 “Click” Reactions in cellulo

Despite of positive results of PROTACs in clinical trials, the development of degraders still shows several challenges. First at all, the physicochemical properties barely fulfill druglikeness rules, like the Lipinski Rule of Five (Ro5).^[91]^ Due to the high molecular weight and polar surface area (800–1000 Da and ∼200 Å^2^, respectively), PROTACs may encounter issues such as scarce cell permeability, low bioavailability, and poor water solubility.[^50^, ^92^, ^93^] Therefore, strategies to overpass these problems and allowing a proper PROTAC delivery inside cells are being developed.[^51^, ^54^] In the following sections, we present selected biorthogonal chemistry examples of these novel approaches, where two click precursors undergo rapid and reversible covalent assembly *in situ*.

### 3.1 Inverse electron demand Diels‐Alder (IEDDA) cycloaddition

One of the most promising approaches is *in‐cell* click‐formed proteolysis targeting chimeras (CLIPTACs). The idea of CLIPTACs was firstly proposed by Astex Pharmaceuticals^[94]^ and it was based on the inverse electron demand Diels‐Alder (IEDDA) cycloaddition between tetrazine and TCO.^[27]^

As the POI ligand, the well‐known bromodomain‐containing protein 4 (BRD4) ligand JQ1 was selected,^[95]^ whereas thalidomide was used as the E3 ligase binder. Then, tetrazine‐tagged thalidomide derivatives (**Tz‐thalidomide**) and trans‐cyclo‐octene‐tagged JQ1 (**JQ1‐TCO**) (Figure 16) were designed and synthetized as smaller PROTAC precursors, able to cross cell membranes. Once inside the cells, these precursors promptly reacted and formed the desired JQ1‐CLIPTAC (**31**, Figure 16). This approach was successfully validated in biological assays, where firstly the formation of **31** in solution was confirmed, and then the **31**‐induced degradation of BRD4 in cells was observed.^[94]^ The authors further corroborated this promising strategy by developing effective CLIPTACs directed to the extracellular‐regulated protein kinase 1/2 (ERK1/2).^[94]^ It is worth noting that, in both cases, the preclicked CLIPTACs (prepared outside cells) did not show any cellular degradation effect, probably due to the lack of cell permeability.^[94]^ This further confirmed that the observed degradation resulted from the self‐assembled CLIPTAC following the precursors’ entry into cells. In addition, the tagged E3 ligase precursors might be exploitable in click reactions with any suitably tagged POI ligand, making this approach largely appliable.

**Figure 16:**
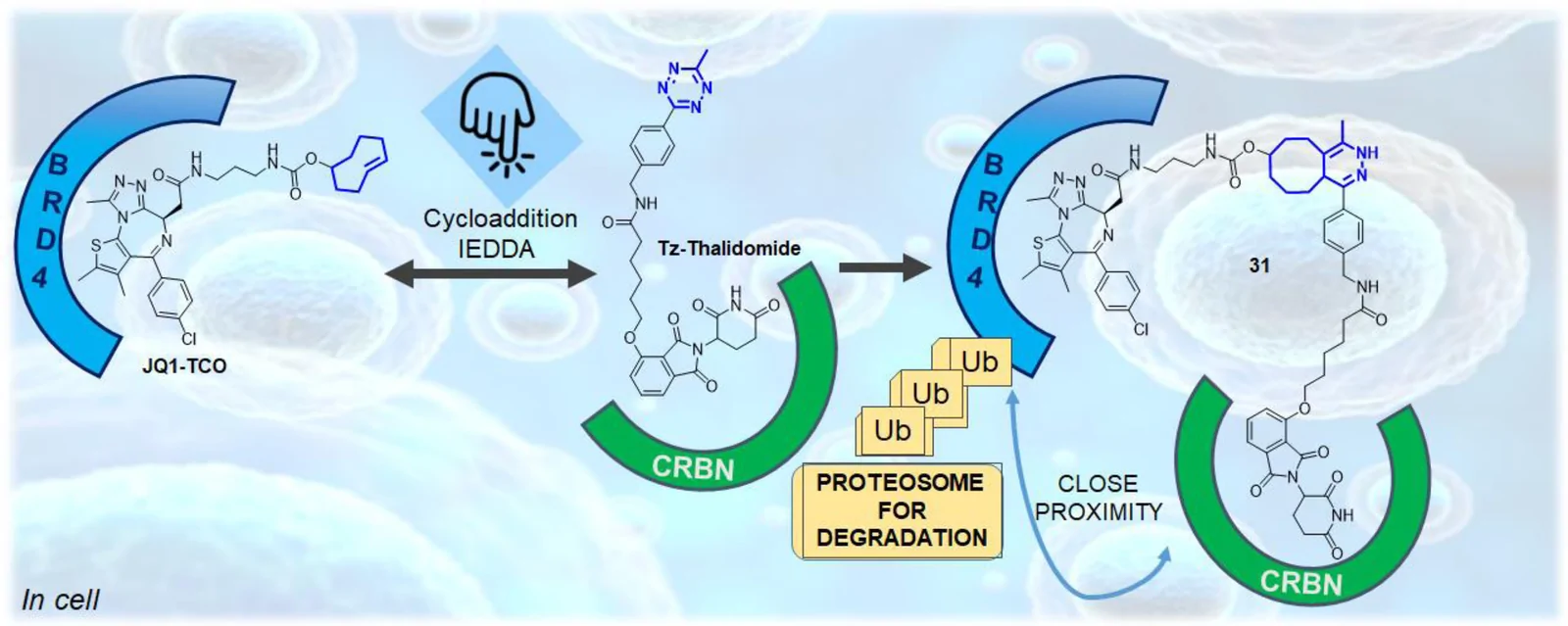
Synthesis of CLIPTAC **31** *via* IEDDA cycloaddition

Indeed, the IEDDA cycloaddition approach has been used in the discovery of novel PROTACs targeting various kinases, including the platelet‐derived growth factor receptor (PDGFR‐β).[^96^, ^97^] Two POI ligands, i. e., linifanib,^[98]^ and S5^[99]^ were modified by introducing the norbornene group and in turn, E3 ligase binders by the tetrazine group (E3L‐Tz). After the confirmation that the self‐assembled PROTACs were formed inside the cells, PDGFR‐β degradation was evaluated by Western blot. The observed effect was not fully satisfactory, probably due to low cell permeability of the E3L‐Tz precursors.^[96]^ Collectively, in spite of significant advances, the development of cell‐permeable PROTACs remains still a challenge.

### 3.2 Copper‐free strain‐promoted azide‐alkyne cycloaddition (SPAAC)

Another bioorthogonal reaction, i. e., the strain‐promoted azide‐alkyne cycloaddition (SPAAC)^[29]^ was exploited for the development of transcription factors (TF) PROTACs (TF‐PROTACs).^[100]^ TF bind specific DNA fragments, and as a result, they control DNA transcription.^[101]^ TF are important biological targets involved in many diseases, especially cancers, but they are considered ‘undruggable’.^[102]^ Therefore, PROTAC technology has successfully been combined with bioorthogonal chemistry, as a promising approach to address these undruggable targets. Liu *et al*. reported a click chemistry platform allowing the fast and easy synthesis of TF‐PROTACs directed to the nuclear factor kappa‐light‐chain‐enhancer of activated B cells (NF‐κB) *via* a copper‐free SPAAC reaction.^[100]^ As E3 ligase binders, a library of eighteen bicyclooctyne (BCN)‐terminated VHL ligands connected to various linkers was synthetized, exemplified by **VHLL‐BCN** carrying a PEG linker (Figure 17). As the NF‐κB binder, a commercially available azide‐modified DNA oligomer (**N_3_‐NF‐κB‐ODN**) was used. The copper‐free SPAAC reactions between **VHLL‐BCN** and **N_3_‐NF‐κB‐ODN** were performed under physiological conditions (PBS, 37 °C), resulting in eighteen TF‐PROTACs, named **dNF‐κBs**, with **32** as the most promising one (Figure 17). Regarding the chemistry part, it is worth noting that, increasing the linker length resulted in lower reaction yields. As for the biological studies, the authors demonstrated that five out of eighteen **dNF‐κBs** reduced p65 protein level. To validate the general utility of this methodology, another series of TF‐PROTACs targeting E2F was successfully developed,^[100]^ demonstrating that bioorthogonal chemistry reactions combined with the PROTAC technology hold great potential also for undruggable targets.

**Figure 17:**
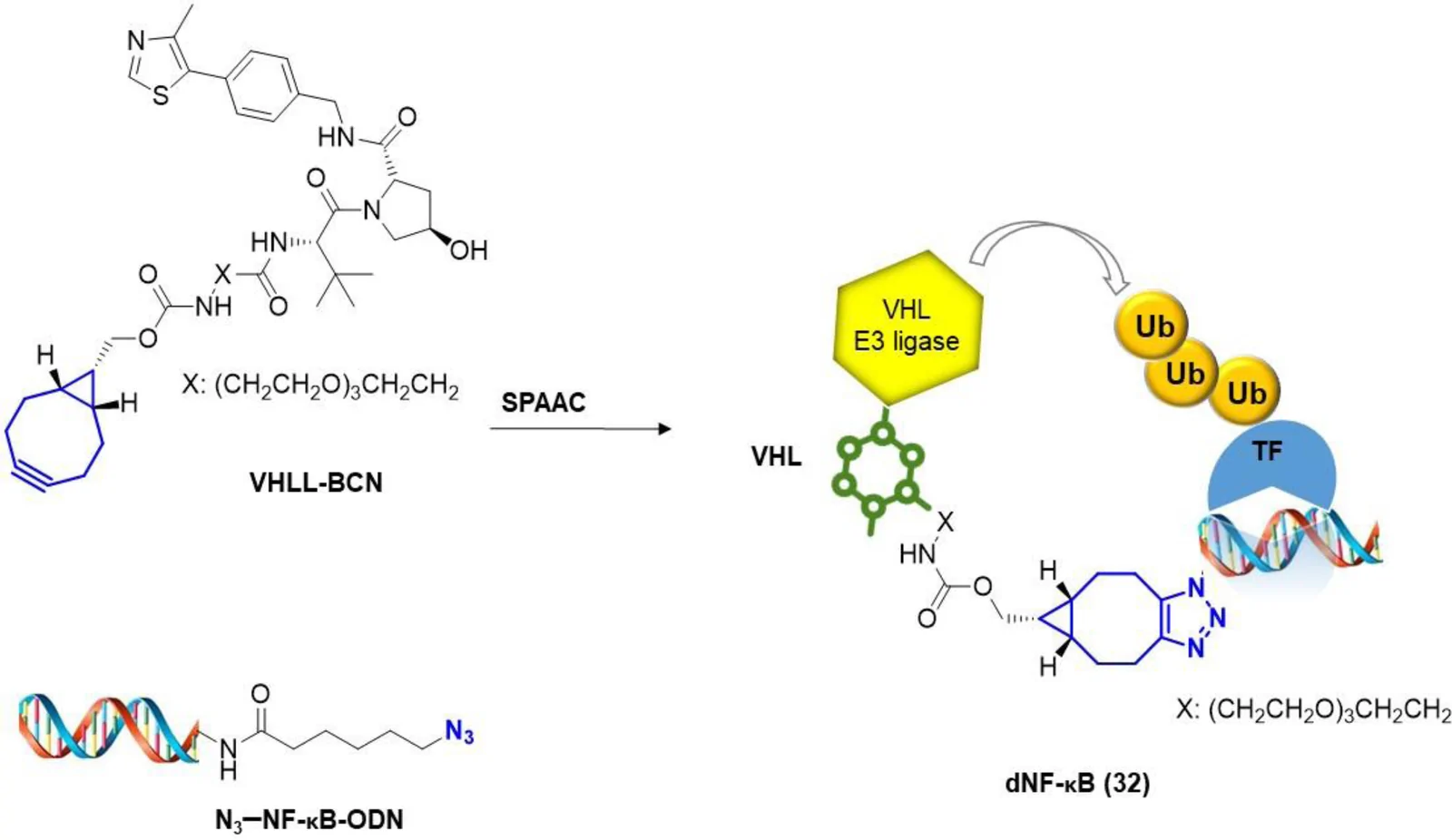
The synthesis of TF‐PROTAC (**32**) *via* a SPAAC reaction.

### 3.3 CuAAC catalyzed by intracellular copper

Si *et al*. proposed another interesting solution to increase the cell permeability and selectivity of anti‐cancer PROTACs, based on a (non‐bioorthogonal) CuAAC *in vivo* catalyzed by endogenous copper (Figure 18).^[103]^ Sorafenib^[104]^ – a multi‐targeted kinase inhibitor, was selected as the POI ligand and was converted into the alkyne terminated precursor (**SA**, Figure 18).

**Figure 18:**
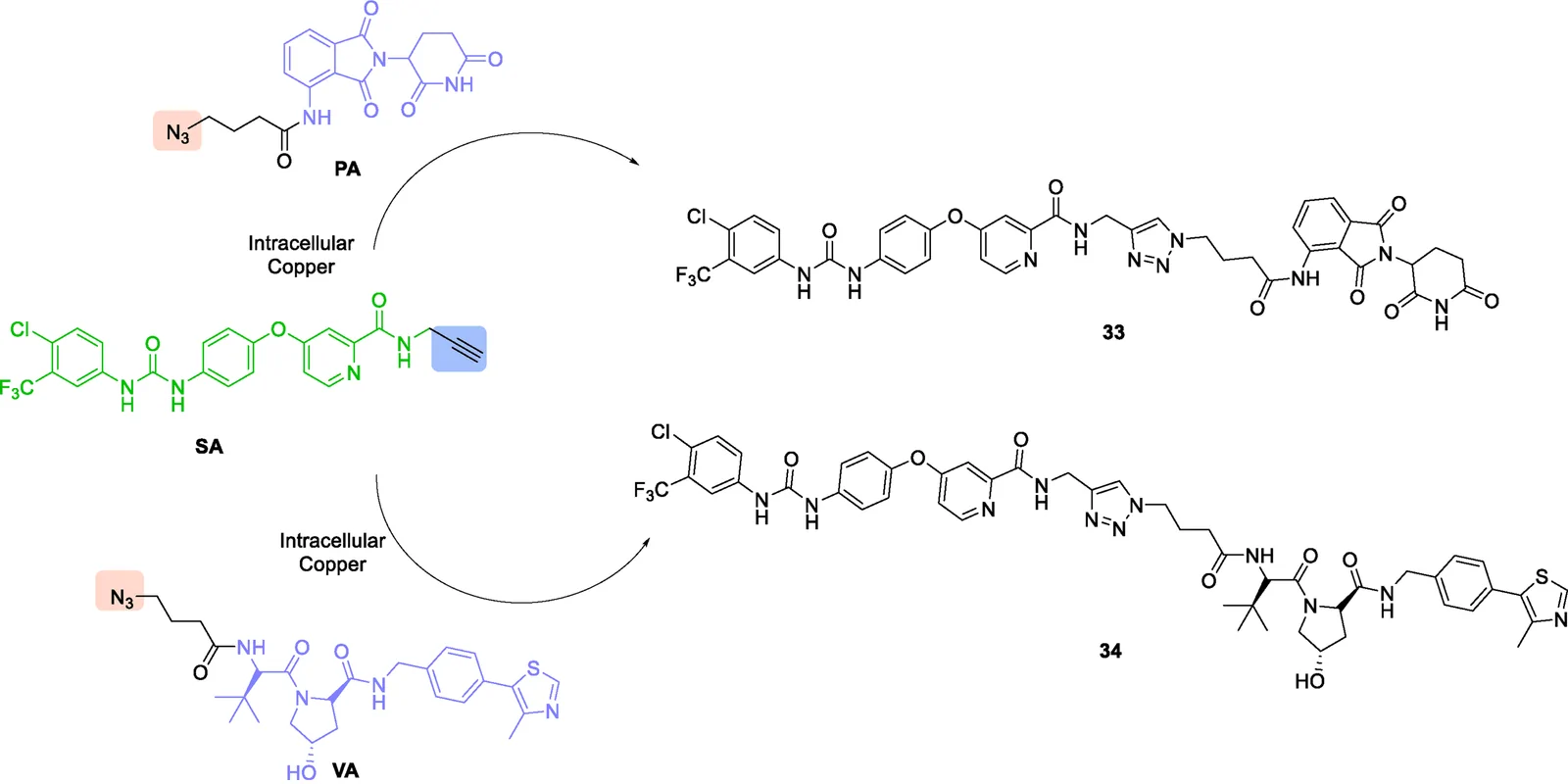
Synthesis anti‐cancer PROTACs **33** and **34** *via* CuAAC reaction *in vivo*.

Azide‐terminated VH032 and pomalidomide ligands (**VA** and **PA**, respectively in Figure 18) were synthesized as E3 ligase binders. The in situ self‐assembly of the two clickable precursors into PROTACs **33** and **34** was catalyzed by the high level of endogenous copper in tumor cells.^[105]^ Once verified the formation of self‐assembled PROTACs – predominately in tumor cells with respect of healthy ones, Western blot analysis showed that only cells treated with **SA** and **VA** remarkably degraded the vascular endothelial growth factor receptor 2 (VEGFR‐2) and EPH receptor B4 (EphB4). The idea of generating tumor‐specific self‐assembled PROTACs by exploiting a click reaction between azides and alkynes together with a higher copper concentration in tumor, might be highly innovative. However, cell permeability and stability of click precursors need to be improved,^[103]^ especially in terms of alternative reactive groups, with more appropriate physicochemical properties.^[54]^

The bioorthogonal reactions are used not only to produce degraders directly in the cells, but also to enhance PROTACs’ bioavailability in specific cells by the development of new delivery techniques, or to control the degradation process. In the following sections, we shortly describe other recent implementations of bioorthogonal reactions for the development of specific degraders.

### 3.4 Bioorthogonal polymeric PROTAC (POLY‐PROTAC) nanoparticles

Gao *et al*. described a polymeric anti‐BRD4 PROTAC nanoplatform to drive selective PROTAC accumulation in tumors.^[106]^ This strategy was based on the preparation of two different nanoparticles (NPs): azide‐terminated NPs containing polymeric PROTAC (POLY‐PROTAC) and DBCO‐modified pretargeted NPs responsible for the tumor delivery. *In situ* bioorthogonal click reaction between these clickable groups enhanced intratumor accumulation and retention of azide‐modified POLY‐PROTAC NPs. Upon internalization into tumor cells, the POLY‐PROTAC NPs released the PROTAC payload *via* a reductive/acidic cleavage. The released VHL‐based PROTACs led to BRD4 degradation and anti‐tumor effect in mice.^[106]^ This study might provide a versatile nanoplatform for enhancing tumor‐specific PROTAC delivery.

### 3.5 Gold nanocluster PROTACs (GNCTACs)

Instead of NPs, Wang *et al*. proposed the use of gold nanoclusters (GNCs) to increase PROTAC bioavailability, especially for peptide‐based PROTACs.[^107^, ^108^] A hybrid nanosized PROTAC (GNCTAC) was prepared by combining GNCs to a human epidermal growth factor receptor 2 (HER2) targeting peptide through gold–sulfur coordination. Then, GNCs were linked to a conjugation unit terminating with a DBCO moiety, which was combined to a CRBN ligand derivatized with an azide group *via* a SPAAC reaction. GNCTAC could efficiently accumulate at the tumor site and efficiently degrade HER2, as well as inhibit tumor growth *in vivo*.^[108]^ Collectively, GNCTACs could overcome the intrinsic limitations of peptide‐based PROTACs (i. e., poor cell permeability and low stability), by efficiently delivering them into cells.

### 3.6 “Ligation to scavenging” approach

Jin *et al*. applied the biorthogonal reactions to the termination of the TPD process.^[109]^ This innovative strategy termed “ligation to scavenging”, aimed to terminate the degradation thanks to the formation of the corresponding triazole‐based inactive PROTACs and to eliminate rapidly them from living cells. The inactivation of PROTACs resulted from the IEDDA reaction between tetrazine‐modified PROTACs (Tz‐PROTACs) and TCO‐modified polyamidoamine dendrimer^[110]^ (PAMAM‐G5‐TCO). This approach was successfully applied to terminate PARP degradation in living cells.^[109]^ Thus, this study provided a valuable approach to regulate PROTAC levels in living cells, which paves the way for a “controlled” TPD.

## 4 Sulfur(VI) Fluoride Exchange (SuFEx) Approach

### 4.1 SuFEx‐enabled high‐throughput medicinal chemistry

The easy and rapidity of SuFEx reaction was implemented in the development of a PROTAC targeting the transcriptional coactivator ENL,^[111]^ involved in the pathogenesis of acute myeloid leukemia (AML).^[112]^ Garnar‐Wortzel *et al*. established an innovative high‐throughput medicinal chemistry approach that leverages SuFEx reactivity to expedite chemical diversification of a starting ENL inhibitor hit.^[111]^ Firstly, the high‐throughput screening (HTS) of a big collection of small molecules led to the identification of an amido‐imidazopyridine ENL inhibitor hit (**HTS hit**, Figure 19). Then, the high‐throughput hit‐to‐lead process relied on parallel synthesis of hit analogs via SuFEx.^[113]^ The biocompatible character of SuFEx transformations allowed that the crude sulfonamide products could be tested directly in biological assays without further purification, thus expediting the medicinal chemistry process. Thanks to this approach, a potent and selective inhibitor **SR‐0813** was discovered (Figure 19). The last step was the synthesis of the first‐in‐class PROTAC targeting transcriptional coactivator ENL, **SR‐1114** (Figure 19), which consists of **SR‐0813** as the POI ligand, a PEG linker and a thalidomide moiety as the E3 ligase binder (Figure 19). The biological assays demonstrated its potential activity in AML and therefore, the success of the SuFEx‐based approach to enable high‐throughput medicinal chemistry, as well as its compatibility with cell‐based drug discovery.^[111]^

**Figure 19:**
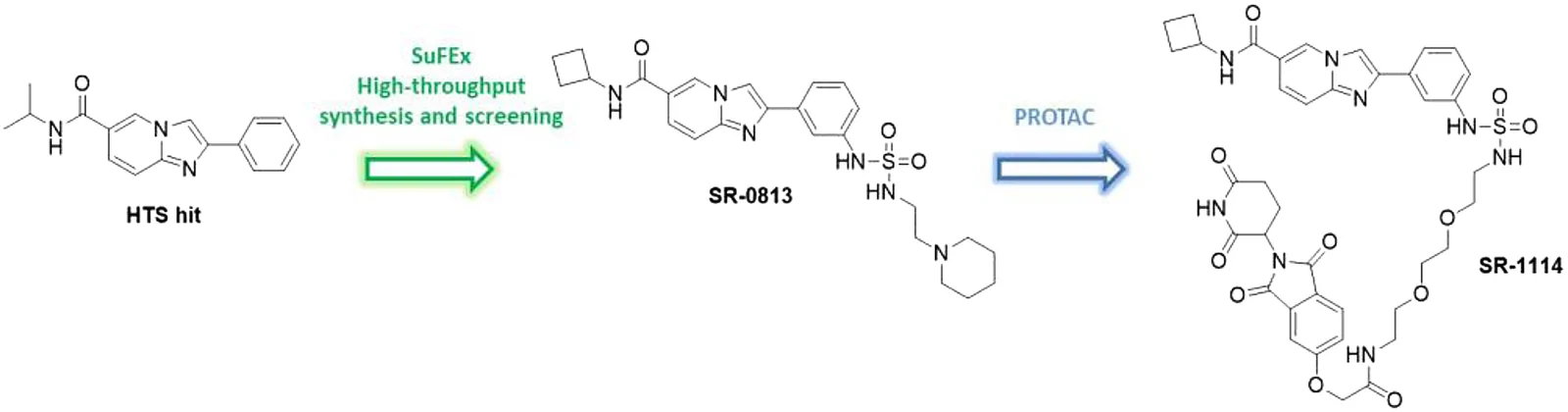
The identification of ENL PROTAC **SR‐1114** using SuFEx‐enabled high‐throughput synthesis and screening.

### 4.2 SuFEx platform for E3 ligase binders

Liu *et al*. applied the SuFEx chemistry for the easy preparation of azide‐terminated E3 ligase binders to be then used in the synthesis of clickable PROTACs.^[114]^ In this case, the fluorosulfuryl azide (FSO_2_N_3_) was used as a diazoting reagent to provide rapid access to the azido derivatives, starting from easily accessible amine precursors. Thus, in a one‐step reaction, a previously developed library of amine‐based immunomodulatory drugs (IMiDs)^[115]^ was converted to azide‐terminated molecules using a procedure reported by Dong and Sharpless (Figure 20A).^[116]^ FSO_2_N_3_ was reacted in a mixture of solvents: methyl *tert*‐butyl ether (MTBE), DMF and water with the addition of KHCO_3_. After few minutes, nineteen derivatives of pomalidomide and lenalidomide with different alkyl or PEG linkers were obtained, with yields between 45 % and 80 %.^[114]^

**Figure 20:**
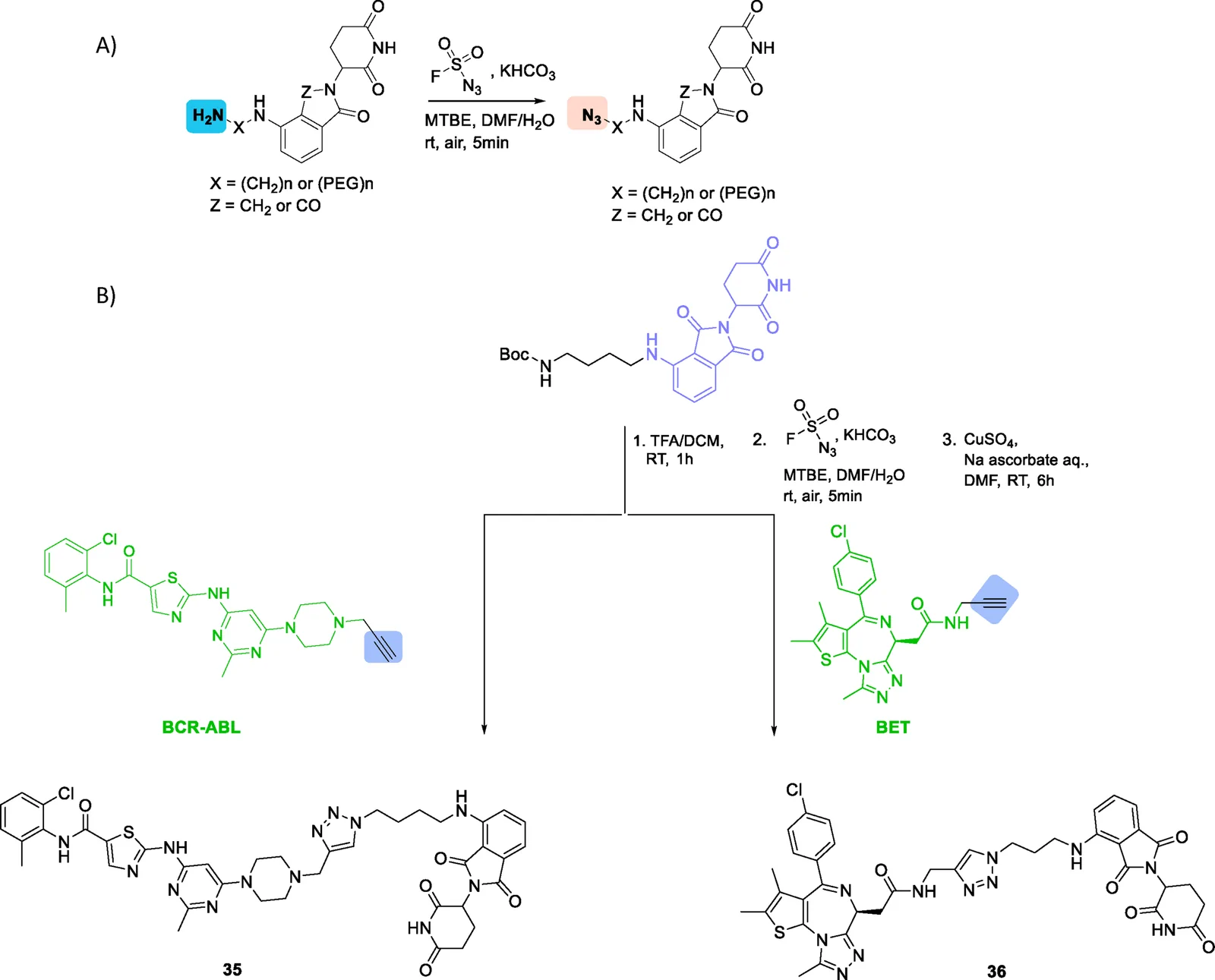
A) One‐step reaction to obtain the azide‐terminated library of IMiDs according to Dong's method; B) three‐step one‐pot process for the synthesis of BCR‐ABL PROTAC **35** and BCR‐ABL PROTAC **36**.

To corroborate the versatility of the synthesized IMiD‐based azide library, PROTACs targeting two different oncogenic proteins, i. e., BCR‐ABL (bromodomain) and BET (extraterminal domain) were prepared. In both cases, previously reported POI ligands[^117^, ^118^] were transformed to alkynyl‐tagged derivatives and then, PROTACs were synthetized *via* a CuAAC reaction (**35** and **36**, Figure 20B). To note, the authors optimized a successful three‐step one‐pot process, including BOC‐deprotection, azide formation and click reaction,^[114]^ with 50–65 % yields (Figure 20B). It is worth mentioning that the newly synthesized BCR‐ABL and BET PROTACs displayed a stronger degradation effect than reference compounds.[^117^, ^118^] Ultimately, the developed IMiD‐based azide library may serve as a platform for speeding up the construction of large and diverse PROTAC libraries.

## 5 Summary and Outlook

TPD is a rapidly emerging modality in medicinal chemistry and drug discovery. A degrader is the prototypical example of a heterobifunctional molecule, constituted by a ligand for the target of interest bridged to a scaffold recruiting the degradation system by an appropriate linker. Therefore, medicinal chemists have struggled to rapidly and effectively assemble the different PROTAC elements in a single molecule able to induce the formation of a productive ternary complex. As a result, the Nobel Prize‐winning technology of click/bioorthogonal chemistry may be a valuable option to the multi‐step synthesis and difficult purification of degraders. The selectivity, ease, rapidity, and modularity of click transformations may speed up degrader synthesis and allow rapid SAR exploration in terms of ligand assembly, attachment points, and linker length/composition.

However, an effective degrader molecule must also possess optimal drug‐like properties. So far, it is generally recognized that a limitation of degraders towards clinical translation is their poor drug‐like profile, mainly due to the low cell permeability and water solubility. To obtain the right balance between potency and drug‐like properties, the exploitation of 1,2,3‐triazole as an optimal linker in PROTAC development, has attracted significant interest. Its introduction could greatly influence the whole physicochemical properties and rigidity of the degrader. Hence, it may be a suitable strategy to improve cell permeability, solubility, or stability. Here, we provided an overview of the combination of TPD technology with click and bioorthogonal chemistry. We discussed selected examples of click chemistry transformations for the development of both small molecule‐ and oligonucleotide‐based PROTACs. This strategy has been also extended to solid‐phase synthesis and combinatorial click chemistry. The possibility to apply these reactions directly *in cellulo* expanded even more the scope and advantages of click chemistry, especially in terms of efficient delivery. Therefore, an avenue that could be pursued to quickly generate PROTACs endowed with optimal drug‐like properties is offered by the cheap and easy click/bioorthogonal chemistry.

Clearly, it is not a panacea. A first issue that has not been enough explored so far is the metabolism of these click‐generated degraders. It is well‐known that triazole‐containing drugs, like fungicides, undergo to a rapid cytochrome P450‐mediated metabolism. This would affect bioavailability and clinical efficacy. In addition, as CYP substrates and inhibitors, triazoles are reported to interact with many drugs, which may lead to toxicity in case of polypharmacy or ineffective treatment.

Furthermore, from a patent perspective, as for September 2023, not so many triazole‐based degraders have been patented. A SciFinder site search for patents containing the keywords “click chemistry” and “PROTAC” retrieved just 6 entries.

All in all, far from being an easy task, we are confident that TPD modality together with click/bioorthogonal chemistry may be a winning combination toward clinical translation.

## Conflict of interest

The authors declare no conflict of interest.

## Biographical Information

*Anna Pasieka obtained her PhD degree with distinction in Pharmaceutical Sciences under the supervision of Professor Barbara Malawska in October 2021 in Jagiellonian University in Kraków. In her PhD thesis she was focused on new multifunctional ligands with potential activity in Alzheimer's disease. During PhD studies, she was awarded a COST STSM within the “MuTaLig” Action spending three months at University of Barcelona. After graduation, she worked as medicinal chemist in the biopharmaceutical company in Warsaw. Since November 2022 Anna is a postdoc in the prof. Bolognesi's group and works in the research project “PROLEISH (PROTACs to treat leishmaniasis)”*.

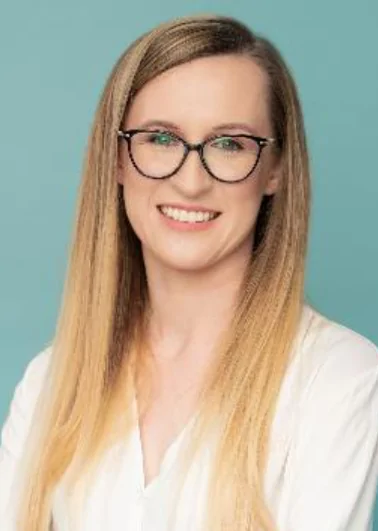

## Biographical Information

*Eleonora Diamanti was trained as medicinal chemist and did her Ph.D. with Prof. Piomelli (IIT, Genova) working one year with Prof. Aggarwal in Bristol on the total synthesis of a natural product to treat Mycobacterium tuberculosis. In 2016, she joined the Hirsch group as a postdoctoral fellow at the University of Groningen and she then moved to the Helmholtz institute (HIPS, Saarbrücken) focusing on the design and synthesis of anti‐infective agents. Since 2023, she is Assistant Professor at the University of Bologna working on the development of PROTAC to combat the antimicrobial resistance problem*.

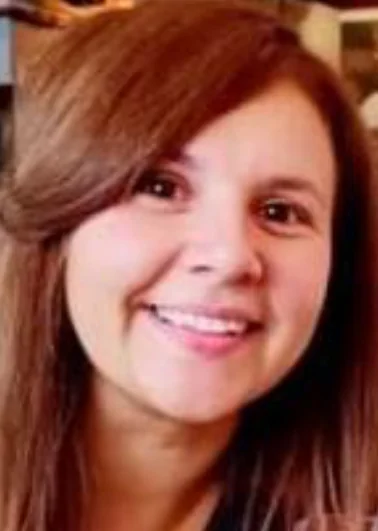

## Biographical Information

*Elisa Uliassi received her M.Sc. degree in Pharmaceutical Chemistry and Technology in 2012, and her Ph.D. in Chemistry in 2016, from the University of Bologna. During her Ph.D. program, she was a visiting Ph.D. student in Prof. L. A. Romeiro's laboratory at Universidade Católica de Brasília, and then in Dr. K. A. Jacobson's laboratory at the National Institutes of Health (U.S.). She was a Visiting Researcher in Prof. M. J. Fuchter's laboratory at the Imperial College of London in 2021. She is currently an Assistant Professor at the Department of Pharmacy and Biotechnology of the University of Bologna*.

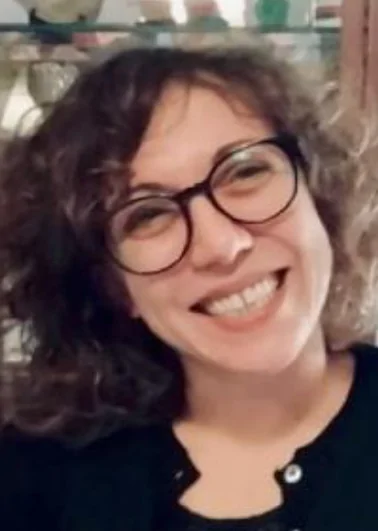

## Biographical Information

*Maria Laura Bolognesi is Professor of Medicinal Chemistry, Coordinator of the Biotechnological, Biocomputational, Pharmaceutical and Pharmacological Sciences PhD Program at the University of Bologna. Her research is concerned with the design, synthesis, and biological investigation of chimeric small molecules in the neurodegenerative and neglected tropical disease therapeutic areas. She has a track record of 200 publications in high‐ranked scientific journals, including patents and patent applications, and numerous invited talks worldwide*.

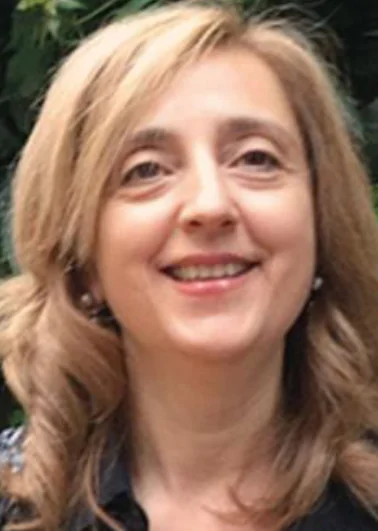

## References

[1] V. V. Rostovtsev , L. G. Green , V. V. Fokin , K. B. Sharpless , Angew. Chem. Int. Ed. 2002, 41, 2596–2599. 10.1002/1521-3773(20020715)41:14<2596::AID-ANIE2596>3.0.CO;2-4 12203546

[2] C. W. Tornøe , C. Christensen , M. Meldal , J. Org. Chem. 2002, 67, 3057–3064. 10.1021/jo011148j 11975567

[3] C. Bertozzi , ACS Cent. Sci. 2022, 2–3.

[4] E. Saxon , C. R. Bertozzi , Science 2000, 287, 2007–2010. 10.1126/science.287.5460.2007 10720325

[5] J. Hou , X. Liu , J. Shen , G. Zhao , P. G. Wang , Expert Opin. Drug Discovery 2012, 7, 489–501. 10.1517/17460441.2012.682725 22607210

[6] H. C. Kolb , K. B. Sharpless , Drug Discovery Today 2003, 8, 401–422.

[7] X. Jiang , X. Hao , L. Jing , G. Wu , D. Kang , X. Liu , P. Zhan , Expert Opin. Drug Discovery 2019, 14, 779–789. 10.1080/17460441.2019.1614910 31094231

[8] H. C. Kolb , M. G. Finn , K. B. Sharpless , Angew. Chem. Int. Ed. 2001, 40, 2004–2021. 10.1002/1521-3773(20010601)40:11<2004::AID-ANIE2004>3.0.CO;2-5 11433435

[9] M. Meldal , C. W. Tomøe , Chem. Rev. 2008, 108, 2952–3015. 10.1021/cr0783479 18698735

[10] C. E. Hoyle , C. N. Bowman , Angew. Chem. Int. Ed. Engl. 2010, 49, 1540–1573. 10.1002/anie.200903924 20166107

[11] Y. Méndez , A. V. Vasco , A. R. Humpierre , B. Westermann , ACS Omega 2020, 5, 25505–25510. 10.1021/acsomega.0c03728 PMC7557220 33073077

[12] J. Tu , D. Svatunek , S. Parvez , A. C. Liu , B. J. Levandowski , H. J. Eckvahl , R. T. Peterson , K. N. Houk , R. M. Franzini , Angew. Chem. Int. Ed. Engl. 2019, 58, 9043–9048. 10.1002/anie.201903877 PMC6615965 31062496

[13] J. Dong , L. Krasnova , M. G. Finn , K. Barry Sharpless , Angew. Chem. Int. Ed. 2014, 53, 9430–9448. 10.1002/anie.201309399 25112519

[14] D. Zeng , W. P. Deng , X. Jiang , Chem. A Eur. J. 2023, 29, e2023005.

[15] T. Guo , G. Meng , X. Zhan , Q. Yang , T. Ma , L. Xu , K. B. Sharpless , J. Dong , Angew. Chemie-Int. Ed. 2018, 57, 2605–2610. 10.1002/anie.201712429 29276888

[16] H.-L. Qin , Q. Zheng , G. A. L. Bare , P. Wu , K. B. Sharpless , Angew. Chem. Int. Ed. Engl. 2016, 55, 14155–14158. 10.1002/anie.201608807 PMC5102157 27723200

[17] Z. Liu , J. Li , S. Li , G. Li , K. B. Sharpless , P. Wu , J. Am. Chem. Soc. 2018, 140, 2919–2925. 10.1021/jacs.7b12788 PMC6192542 29451783

[18] C. Zhao , K. P. Rakesh , L. Ravidar , W.-Y. Fang , H.-L. Qin , Eur. J. Med. Chem. 2019, 162, 679–734. 10.1016/j.ejmech.2018.11.017 PMC7111228 30496988

[19] A. S. Barrow , C. J. Smedley , Q. Zheng , S. Li , J. Dong , J. E. Moses , Chem. Soc. Rev. 2019, 48, 4731–4758. 10.1039/c8cs00960k 31364998

[20] S. Wilson Lucas , R. Zijian Qin , K. P. Rakesh , K. S. Sharath Kumar , H.-L. Qin , Bioorg. Chem. 2023, 130, 106227. 10.1016/j.bioorg.2022.106227 36368173

[21] E. M. Sletten , C. R. Bertozzi , Acc. Chem. Res. 2011, 44, 666–676. 10.1021/ar200148z PMC3184615 21838330

[22] J. C. Jewett , C. R. Bertozzi , Chem. Soc. Rev. 2010, 39, 1272–1279. 10.1039/b901970g PMC2865253 20349533

[23] K. L. Kiick , E. Saxon , D. A. Tirelli , C. R. Bertozzi , PNAS 2002, 99, 19–24. 10.1073/pnas.012583299 PMC117506 11752401

[24] V. O. Rodionov , V. V. Fokin , M. G. Finn , Angew. Chem. Int. Ed. 2005, 44, 2210–2215. 10.1002/anie.200461496 15693051

[25] B. L. Oliveira , Z. Guo , G. J. L. Bernardes , Chem. Soc. Rev. 2017, 46, 4895–4950. 10.1039/c7cs00184c 28660957

[26] J.-B. Béquignat , N. Ty , A. Rondon , L. Taiariol , F. Degoul , D. Canitrot , M. Quintana , I. Navarro-Teulon , E. Miot-Noirault , C. Boucheix , J.-M. Chezal , E. Moreau , Eur. J. Med. Chem. 2020, 203, 112574. 10.1016/j.ejmech.2020.112574 32683167

[27] M. L. Blackman , M. Royzen , J. M. Fox , J. Am. Chem. Soc. 2008, 130, 13518–13519. 10.1021/ja8053805 PMC2653060 18798613

[28] C. Bednarek , I. Wehl , N. Jung , U. Schepers , S. Bräse , Chem. Rev. 2020, 120, 4301–4354. 10.1021/acs.chemrev.9b00665 32356973

[29] N. J. Agard , J. A. Prescher , C. R. Bertozzi , J. Am. Chem. Soc. 2004, 126, 15046–15047. 10.1021/ja044996f 15547999

[30] M. Handula , K.-T. Chen , Y. Seimbille , Molecules 2021, 26, 10.3390/molecules26154640. PMC8347371 34361793

[31] K. Bozorov , J. Zhao , H. A. Aisa , Bioorg. Med. Chem. 2019, 27, 3511–3531. 10.1016/j.bmc.2019.07.005 PMC7185471 31300317

[32] N. K. Devaraj , ACS Cent. Sci. 2018, 4, 952–959. 10.1021/acscentsci.8b00251 PMC6107859 30159392

[33] The Nobel Committee for Chemistry, *K. Vetenkaps-Akademien* **2022**, *50005*, 1–18.

[34] T. W. Corson , N. Aberle , C. M. Crews , ACS Chem. Biol. 2008, 3, 677–692. 10.1021/cb8001792 PMC2925120 19112665

[35] A. C. Lai , C. M. Crews , N. Haven , Nat. Rev. Drug Discov. (84.69; Q1) 2017, 16, 101–114. 10.1038/nrd.2016.211 PMC5684876 27885283

[36] X. Sun , H. Gao , Y. Yang , M. He , Y. Wu , Y. Song , Y. Tong , Y. Rao , Signal Transduct. Target. Ther. 2019, 4, 10.1038/s41392-019-0101-6. PMC6927964 31885879

[37] M. He , C. Cao , Z. Ni , Y. Liu , P. Song , S. Hao , Y. He , X. Sun , Y. Rao , Signal Transduct. Target. Ther. 2022, 7, 10.1038/s41392-022-00999-9. PMC9178337 35680848

[38] G. Ahn , S. M. Banik , C. L. Miller , N. M. Riley , J. R. Cochran , C. R. Bertozzi , Nat. Chem. Biol. 2021, 17, 937–946. 10.1038/s41589-021-00770-1 PMC8387313 33767387

[39] J. Pei , X. Pan , A. Wang , W. Shuai , F. Bu , P. Tang , S. Zhang , Y. Zhang , G. Wang , L. Ouyang , Chem. Commun. 2021, 57, 13194–13197. 10.1039/d1cc04661f 34816823

[40] D. Takahashi , J. Moriyama , T. Nakamura , E. Miki , E. Takahashi , A. Sato , T. Akaike , K. Itto-Nakama , H. Arimoto , Mol. Cell 2019, 76, 797–810.e10. 10.1016/j.molcel.2019.09.009 31606272

[41] S. B. Alabi , C. M. Crews , J. Biol. Chem. 2021, 296, 100647. 10.1016/j.jbc.2021.100647 PMC8131913 33839157

[42] L. Hua , Q. Zhang , X. Zhu , R. Wang , Q. You , L. Wang , J. Med. Chem. 2022, 65, 8091–8112. 10.1021/acs.jmedchem.2c00316 35686733

[43] A. Salerno , F. Seghetti , J. Caciolla , E. Uliassi , E. Testi , M. Guardigni , M. Roberti , A. Milelli , M. L. Bolognesi , J. Med. Chem. 2022, 65, 9507–9530. 10.1021/acs.jmedchem.2c00302 PMC9340767 35816671

[44] R. M. Espinoza-Chávez , A. Salerno , A. Liuzzi , A. Ilari , A. Milelli , E. Uliassi , M. L. Bolognesi , ACS bio med chem Au 2023, 3, 32–45. 10.1021/acsbiomedchemau.2c00063 PMC10125329 37101607

[45] M. Teng , N. S. Gray , Cell Chem. Biol. 2023, 30, 10.1016/j.chembiol.2023.06.020. 37494935

[46] X. Liu , A. Ciulli , ACS Cent. Sci. 2023, 9, 1269–1284. 10.1021/acscentsci.3c00395 PMC10375889 37521793

[47] C. Steinebach , I. Sosič , S. Lindner , A. Bricelj , F. Kohl , Y. L. D. Ng , M. Monschke , K. G. Wagner , J. Krönke , M. Gütschow , MedChemComm 2019, 10, 1037–1041. 10.1039/c9md00185a PMC6596386 31304001

[48] M. S. Gadd , A. Testa , X. Lucas , K.-H. Chan , W. Chen , D. J. Lamont , M. Zengerle , A. Ciulli , Nat. Chem. Biol. 2017, 13, 514–521. 10.1038/nchembio.2329 PMC5392356 28288108

[49] M. J. Roy , S. Winkler , S. J. Hughes , C. Whitworth , M. Galant , W. Farnaby , K. Rumpel , A. Ciulli , ACS Chem. Biol. 2019, 14, 361–368. 10.1021/acschembio.9b00092 PMC6423499 30721025

[50] H. Lebraud , T. D. Heightman , Essays Biochem. 2017, 61, 517–527. 10.1042/EBC20170030 28970340

[51] V. G. Klein , C. E. Townsend , A. Testa , M. Zengerle , C. Maniaci , S. J. Hughes , K.-H. Chan , A. Ciulli , R. S. Lokey , ACS Med. Chem. Lett. 2020, 11, 1732–1738. 10.1021/acsmedchemlett.0c00265 PMC7488288 32939229

[52] A. Zagidullin , V. Milyukov , A. Rizvanov , E. Bulatov , Explor. Target. Anti-tumor Ther. 2020, 1, 381–390. 10.37349/etat.2020.00023 PMC9400696 36046487

[53] N. Bai , S. A. Miller , G. V. Andrianov , M. Yates , P. Kirubakaran , J. Karanicolas , J. Chem. Inf. Model. 2021, 61, 1368–1382. 10.1021/acs.jcim.0c01451 PMC8866032 33625214

[54] S. Tomoshige , M. Ishikawa , Bioorg. Med. Chem. 2021, 41, 116221. 10.1016/j.bmc.2021.116221 34034148

[55] C. M. Crews , J. Med. Chem. 2018, 61, 403–404. 10.1021/acs.jmedchem.7b01333 29164885

[56] M. Schiedel , D. Herp , S. Hammelmann , S. Swyter , A. Lehotzky , D. Robaa , J. Oláh , J. Ovádi , W. Sippl , M. Jung , J. Med. Chem. 2018, 61, 482–491. 10.1021/acs.jmedchem.6b01872 28379698

[57] Q.-J. Wu , T.-N. Zhang , H.-H. Chen , X.-F. Yu , J.-L. Lv , Y.-Y. Liu , Y.-S. Liu , G. Zheng , J.-Q. Zhao , Y.-F. Wei , J.-Y. Guo , F.-H. Liu , Q. Chang , Y.-X. Zhang , C.-G. Liu , Y.-H. Zhao , Signal Transduct. Target. Ther. 2022, 7, 402. 10.1038/s41392-022-01257-8 PMC9797940 36581622

[58] T. Rumpf , M. Schiedel , B. Karaman , C. Roessler , B. J. North , A. Lehotzky , J. Oláh , K. I. Ladwein , K. Schmidtkunz , M. Gajer , M. Pannek , C. Steegborn , D. A. Sinclair , S. Gerhardt , J. Ovádi , M. Schutkowski , W. Sippl , O. Einsle , M. Jung , Nat. Commun. 2015, 6, 6263. 10.1038/ncomms7263 PMC4339887 25672491

[59] R. P. Wurz , K. Dellamaggiore , H. Dou , N. Javier , M. C. Lo , J. D. McCarter , D. Mohl , C. Sastri , J. R. Lipford , V. J. Cee , J. Med. Chem. 2018, 61, 453–461. 10.1021/acs.jmedchem.6b01781 28378579

[60] P. Filippakopoulos , J. Qi , S. Picaud , Y. Shen , W. B. Smith , O. Fedorov , E. M. Morse , T. Keates , T. T. Hickman , I. Felletar , M. Philpott , S. Munro , M. R. McKeown , Y. Wang , A. L. Christie , N. West , M. J. Cameron , B. Schwartz , T. D. Heightman , N. La Thangue , C. A. French , O. Wiest , A. L. Kung , S. Knapp , J. E. Bradner , Nature 2010, 468, 1067–1073. 10.1038/nature09504 PMC3010259 20871596

[61] L. Wang , X. Shao , T. Zhong , Y. Wu , A. Xu , X. Sun , H. Gao , Y. Liu , T. Lan , Y. Tong , X. Tao , W. Du , W. Wang , Y. Chen , T. Li , X. Meng , H. Deng , B. Yang , Q. He , M. Ying , Y. Rao , Nat. Chem. Biol. 2021, 17, 567–575. 10.1038/s41589-021-00742-5 33664520

[62] H. Döhner , D. J. Weisdorf , C. D. Bloomfield , N. Engl. J. Med. 2015, 373, 1136–1152. 10.1056/NEJMra1406184 26376137

[63] O. R. Ayinde , C. Sharpe , E. Stahl , R. J. T. Ii , J. R. Lerma , N. Muthusamy , J. C. Byrd , J. R. Fuchs , ACS Med. Chem. Lett. 2023, DOI 10.1021/acsmedchemlett.3c00082. 10.1021/acsmedchemlett.3c00082 PMC10351057 37465296

[64] M. S. Squires , R. E. Feltell , N. G. Wallis , E. J. Lewis , D.-M. Smith , D. M. Cross , J. F. Lyons , N. T. Thompson , Mol. Cancer Ther. 2009, 8, 324–332. 10.1158/1535-7163.MCT-08-0890 19174555

[65] L. Qu , S. Li , L. Ji , S. Luo , M. Ding , F. Yin , C. Wang , H. Luo , D. Lu , X. Liu , W. Peng , L. Kong , X. Wang , Eur. J. Med. Chem. 2021, 226, 10.1016/j.ejmech.2021.113889. 34649182

[66] X. Jiang , J. Zhou , Y. Wang , X. Liu , K. Xu , J. Xu , F. Feng , H. Sun , Eur. J. Med. Chem. 2021, 210, 112949. 10.1016/j.ejmech.2020.112949 33097303

[67] P. Sivaprakasam , X. Han , R. L. Civiello , S. Jacutin-Porte , K. Kish , M. Pokross , H. A. Lewis , N. Ahmed , N. Szapiel , J. A. Newitt , E. T. Baldwin , H. Xiao , C. M. Krause , H. Park , M. Nophsker , J. S. Lippy , C. R. Burton , D. R. Langley , J. E. Macor , G. M. Dubowchik , Bioorg. Med. Chem. Lett. 2015, 25, 1856–1863. 10.1016/j.bmcl.2015.03.046 25845281

[68] K. Yang , Y. Song , H. Xie , H. Wu , Y. T. Wu , E. D. Leisten , W. Tang , Bioorg. Med. Chem. Lett. 2018, 28, 2493–2497. 10.1016/j.bmcl.2018.05.057 29871848

[69] C. Boyault , K. Sadoul , M. Pabion , S. Khochbin , Oncogene 2007, 26, 5468–5476. 10.1038/sj.onc.1210614 17694087

[70] T. Hideshima , J. Qi , R. M. Paranal , W. Tang , E. Greenberg , N. West , M. E. Colling , G. Estiu , R. Mazitschek , J. A. Perry , H. Ohguchi , F. Cottini , N. Mimura , G. Görgün , Y.-T. Tai , P. G. Richardson , R. D. Carrasco , O. Wiest , S. L. Schreiber , K. C. Anderson , J. E. Bradner , Proc. Natl. Acad. Sci. USA 2016, 113, 13162–13167. 10.1073/pnas.1608067113 PMC5135369 27799547

[71] S. Neumann , M. Biewend , S. Rana , W. H. Binder , Macromol. Rapid Commun. 2020, 41, 10.1002/marc.201900359. 31631449

[72] H. Wu , K. Yang , Z. Zhang , E. D. Leisten , Z. Li , H. Xie , J. Liu , K. A. Smith , Z. Novakova , C. Barinka , W. Tang , J. Med. Chem. 2019, 62, 7042–7057. 10.1021/acs.jmedchem.9b00516 31271281

[73] E. Munoz , G. Chen , A. Hossain , S. Wu , E. Oceguera Nava , J. Hang , T. Lee , Q. Zhang , G. Wang , Q. H. Chen , Bioorg. Med. Chem. Lett. 2022, 72, 128870. 10.1016/j.bmcl.2022.128870 PMC9729070 35772635

[74] G. Loren , I. Espuny , A. Llorente , C. Donoghue , X. Verdaguer , R. R. Gomis , A. Riera , Eur. J. Med. Chem. 2022, 243, 114770. 10.1016/j.ejmech.2022.114770 36148710

[75] Y. Chen , X. Yuan , M. Tang , M. Shi , T. Yang , K. Liu , D. Deng , L. Chen , Bioorg. Chem. 2022, 119, 105508. 10.1016/j.bioorg.2021.105508 34959180

[76] S. Su , Z. Yang , H. Gao , H. Yang , S. Zhu , Z. An , J. Wang , Q. Li , S. Chandarlapaty , H. Deng , W. Wu , Y. Rao , J. Med. Chem. 2019, 62, 7575–7582. 10.1021/acs.jmedchem.9b00871 PMC6790125 31330105

[77] M. Zheng , J. Huo , X. Gu , Y. Wang , C. Wu , Q. Zhang , W. Wang , Y. Liu , Y. Liu , X. Zhou , L. Chen , Y. Zhou , H. Li , J. Med. Chem. 2021, 64, 7839–7852. 10.1021/acs.jmedchem.1c00649 34038131

[78] T. K. Neklesa , H. S. Tae , A. R. Schneekloth , M. J. Stulberg , T. W. Corson , T. B. Sundberg , K. Raina , S. A. Holley , C. M. Crews , Nat. Chem. Biol. 2011, 7, 538–543. 10.1038/nchembio.597 PMC3139752 21725302

[79] H. Xie , J. J. Liang , Y. L. Wang , T. X. Hu , J. Y. Wang , R. H. Yang , J. K. Yan , Q. R. Zhang , X. Xu , H. M. Liu , Y. Ke , Eur. J. Med. Chem. 2020, 204, 10.1016/j.ejmech.2020.112512. 32736229

[80] M. E. Van Dort , D. M. Robins , B. Wayburn , J. Med. Chem. 2000, 43, 3344–3347. 10.1021/jm000163y 10966753

[81] H. S. Tae , T. B. Sundberg , T. K. Neklesa , D. J. Noblin , J. L. Gustafson , A. G. Roth , K. Raina , C. M. Crews , ChemBioChem 2012, 13, 538–541. 10.1002/cbic.201100793 PMC3338156 22271667

[82] M. Naganuma , N. Ohoka , G. Tsuji , H. Tsujimura , K. Matsuno , T. Inoue , M. Naito , Y. Demizu , ACS Med. Chem. Lett. 2022, 13, 134–139. 10.1021/acsmedchemlett.1c00629 PMC8762735 35059133

[83] G. M. Burslem , C. M. Crews , Chem. Rev. 2017, 117, 11269–11301. 10.1021/acs.chemrev.7b00077 28777566

[84] X. Zhang , Z. Zhang , X. Xue , T. Fan , C. Tan , F. Liu , Y. Tan , Y. Jiang , ACS Pharmacol. Transl. Sci. 2022, 5, 1109–1118. 10.1021/acsptsci.2c00109 PMC9667539 36407946

[85] C. M. Klinge , Nucleic Acids Res. 2001, 29, 2905–2919. 10.1093/nar/29.14.2905 PMC55815 11452016

[86] J. S. Disch , J. M. Duffy , E. C. Y. Lee , D. Gikunju , B. Chan , B. Levin , M. I. Monteiro , S. A. Talcott , A. C. Lau , F. Zhou , A. Kozhushnyan , N. E. Westlund , P. B. Mullins , Y. Yu , M. Von Rechenberg , J. Zhang , Y. A. Arnautova , Y. Liu , Y. Zhang , A. J. McRiner , A. D. Keefe , A. Kohlmann , M. A. Clark , J. W. Cuozzo , C. Huguet , S. Arora , J. Med. Chem. 2021, 64, 5049–5066. 10.1021/acs.jmedchem.1c00127 33844532

[87] M. A. Clark , R. A. Acharya , C. C. Arico-Muendel , S. L. Belyanskaya , D. R. Benjamin , N. R. Carlson , P. A. Centrella , C. H. Chiu , S. P. Creaser , J. W. Cuozzo , C. P. Davie , Y. Ding , G. J. Franklin , K. D. Franzen , M. L. Gefter , S. P. Hale , N. J. V. Hansen , D. I. Israel , J. Jiang , M. J. Kavarana , M. S. Kelley , C. S. Kollmann , F. Li , K. Lind , S. Mataruse , P. F. Medeiros , J. A. Messer , P. Myers , H. O'Keefe , M. C. Oliff , C. E. Rise , A. L. Satz , S. R. Skinner , J. L. Svendsen , L. Tang , K. van Vloten , R. W. Wagner , G. Yao , B. Zhao , B. A. Morgan , Nat. Chem. Biol. 2009, 5, 647–654. 10.1038/nchembio.211 19648931

[88] N. Cankařová , E. Schütznerová , V. Krchňák , Chem. Rev. 2019, 119, 12089–12207. 10.1021/acs.chemrev.9b00465 31747265

[89] H. Xu , T. Kurohara , R. Takano , H. Yokoo , N. Shibata , N. Ohoka , T. Inoue , M. Naito , Y. Demizu , ChemistryOpen 2022, 11, 1–6. 10.1002/open.202200131 PMC9278092 35822913

[90] S. Krajcovicova , R. Jorda , D. Hendrychova , V. Krystof , M. Soural , Chem. Commun. 2019, 55, 929–932. 10.1039/c8cc08716d 30601480

[91] C. A. Lipinski , F. Lombardo , B. W. Dominy , P. J. Feeney , Adv. Drug Delivery Rev. 2001, 46, 3–26. 10.1016/s0169-409x(00)00129-0 11259830

[92] P. Matsson , J. Kihlberg , J. Med. Chem. 2017, 60, 1662–1664. 10.1021/acs.jmedchem.7b00237 28234469

[93] S. D. Edmondson , B. Yang , C. Fallan , Bioorg. Med. Chem. Lett. 2019, 29, 1555–1564. 10.1016/j.bmcl.2019.04.030 31047748

[94] H. Lebraud , D. J. Wright , C. N. Johnson , T. D. Heightman , ACS Cent. Sci. 2016, 2, 927–934. 10.1021/acscentsci.6b00280 PMC5200928 28058282

[95] M. Zengerle , K.-H. Chan , A. Ciulli , ACS Chem. Biol. 2015, 10, 1770–1777. 10.1021/acschembio.5b00216 PMC4548256 26035625

[96] R. Si , H. Zhu , J. Wang , Q. Zhang , Y. Li , X. Pan , J. Zhang , Bioorg. Chem. 2023, 134, 10.1016/j.bioorg.2023.106439. 36842319

[97] R. Si , H. Zhu , J. Wang , Q. Zhang , Y. Li , X. Pan , J. Zhang , Bioorg. Chem. 2023, 135, 10.1016/j.bioorg.2023.106497. 37003135

[98] J. Chen , J. Guo , Z. Chen , J. Wang , M. Liu , X. Pang , Sci. Rep. 2016, 6, 29382. 10.1038/srep29382 PMC4937412 27387652

[99] Y. Shan , H. Gao , X. Shao , J. Wang , X. Pan , J. Zhang , Eur. J. Med. Chem. 2015, 103, 80–90. 10.1016/j.ejmech.2015.08.045 26342134

[100] J. Liu , H. Chen , H. Ü. Kaniskan , L. Xie , X. Chen , J. Jin , W. Wei , J. Am. Chem. Soc. 2021, 143, 8902–8910. 10.1021/jacs.1c03852 PMC8225582 34100597

[101] S. A. Lambert , A. Jolma , L. F. Campitelli , P. K. Das , Y. Yin , M. Albu , X. Chen , J. Taipale , T. R. Hughes , M. T. Weirauch , Cell 2018, 172, 650–665. 10.1016/j.cell.2018.01.029 PMC12908702 29425488

[102] J. H. Bushweller , Nat. Rev. Cancer 2019, 19, 611–624. 10.1038/s41568-019-0196-7 PMC8820243 31511663

[103] R. Si , P. Hai , Y. Zheng , J. Wang , Q. Zhang , Y. Li , X. Pan , J. Zhang , Eur. J. Med. Chem. 2023, 257, 115497. 10.1016/j.ejmech.2023.115497 37216813

[104] S. M. Wilhelm , C. Carter , L. Y. Tang , D. Wilkie , A. McNabola , H. Rong , C. Chen , X. Zhang , P. Vincent , M. McHugh , Y. Cao , J. Shujath , S. Gawlak , D. Eveleigh , B. Rowley , L. Liu , L. Adnane , M. Lynch , D. Auclair , I. Taylor , R. Gedrich , A. Voznesensky , B. Riedl , L. E. Post , G. Bollag , P. A. Trail , Cancer Res. 2004, 64, 7099–7109. 10.1158/0008-5472.CAN-04-1443 15466206

[105] T. Kamiya , J. Clin. Biochem. Nutr. 2022, 71, 22–28. 10.3164/jcbn.22-9 PMC9309082 35903604

[106] J. Gao , B. Hou , Q. Zhu , L. Yang , X. Jiang , Z. Zou , X. Li , T. Xu , M. Zheng , Y. H. Chen , Z. Xu , H. Xu , H. Yu , Nat. Commun. 2022, 13, 1–14.

[107] Z. Luo , X. Yuan , Y. Yu , Q. Zhang , D. T. Leong , J. Y. Lee , J. Xie , J. Am. Chem. Soc. 2012, 134, 16662–16670. 10.1021/ja306199p 22998450

[108] Z. Wang , M. Tan , W. Su , W. Huang , J. Zhang , F. Jia , G. Cao , X. Liu , H. Song , H. Ran , G. Nie , H. Wang , J. Med. Chem. 2023, 66(9), 6263–6273. 10.1021/acs.jmedchem.3c00013 37092695

[109] Y. Jin , J. Fan , R. Wang , X. Wang , N. Li , Q. You , Z. Jiang , J. Am. Chem. Soc. 2022, 145(13), 7218–7229. 10.1021/jacs.2c12809 36971523

[110] H. Kheraldine , O. Rachid , A. M. Habib , A.-E. Al Moustafa , I. F. Benter , S. Akhtar , Adv. Drug Delivery Rev. 2021, 178, 113908. 10.1016/j.addr.2021.113908 34390777

[111] L. Garnar-Wortzel , T. R. Bishop , S. Kitamura , N. Milosevich , J. N. Asiaban , X. Zhang , Q. Zheng , E. Chen , A. R. Ramos , C. J. Ackerman , E. N. Hampton , A. K. Chatterjee , T. S. Young , M. V. Hull , K. B. Sharpless , B. F. Cravatt , D. W. Wolan , M. A. Erb , ACS Cent. Sci. 2021, 7, 815–830. 10.1021/acscentsci.0c01550 PMC8161486 34079898

[112] M. A. Erb , T. G. Scott , B. E. Li , H. Xie , J. Paulk , H.-S. Seo , A. Souza , J. M. Roberts , S. Dastjerdi , D. L. Buckley , N. E. Sanjana , O. Shalem , B. Nabet , R. Zeid , N. K. Offei-Addo , S. Dhe-Paganon , F. Zhang , S. H. Orkin , G. E. Winter , J. E. Bradner , Nature 2017, 543, 270–274. 10.1038/nature21688 PMC5497220 28241139

[113] S. Kitamura , Q. Zheng , J. L. Woehl , A. Solania , E. Chen , N. Dillon , M. V. Hull , M. Kotaniguchi , J. R. Cappiello , S. Kitamura , V. Nizet , K. B. Sharpless , D. W. Wolan , J. Am. Chem. Soc. 2020, 142, 10899–10904. 10.1021/jacs.9b13652 PMC7751259 32479075

[114] H. Liu , R. Sun , C. Ren , X. Qiu , X. Yang , B. Jiang , Org. Biomol. Chem. 2021, 19, 166–170. 10.1039/d0ob02120b 33226388

[115] X. Qiu , N. Sun , Y. Kong , Y. Li , X. Yang , B. Jiang , Org. Lett. 2019, 21, 3838–3841. 10.1021/acs.orglett.9b01326 31066567

[116] G. Meng , T. Guo , T. Ma , J. Zhang , Y. Shen , K. B. Sharpless , J. Dong , Nature 2019, 574, 86–89. 10.1038/s41586-019-1589-1 31578481

[117] G. E. Winter , D. L. Buckley , J. Paulk , J. M. Roberts , A. Souza , S. Dhe-Paganon , J. E. Bradner , Science 2015, 348, 1376–1381. 10.1126/science.aab1433 PMC4937790 25999370

[118] A. C. Lai , M. Toure , D. Hellerschmied , J. Salami , S. Jaime-Figueroa , E. Ko , J. Hines , C. M. Crews , Angew. Chem. Int. Ed. Engl. 2016, 55, 807–810. 10.1002/anie.201507634 PMC4733637 26593377

